# The Communication Issue in Developing Childcare Services for Children Under Three Years of Age in China: An Analysis of Policy Texts and Practical Cases

**DOI:** 10.3390/healthcare14060776

**Published:** 2026-03-19

**Authors:** Hanxiao Liu, Jianghua Liu

**Affiliations:** The Institute for Population & Development Studies, School of Public Policy & Administration, Xi’an Jiaotong University, No. 28, West Xianning Road, Xi’an 710049, China; liuhanxiao@stu.xjtu.edu.cn

**Keywords:** child health, China, communication, formal childcare, health policy, health promotion, health services, public–private partnership, social network analysis, textual analysis

## Abstract

**Highlights:**

**What are the main findings?**

- The communication of childcare services to prospective parents was not given sufficient attention in governmental policies.
- The activities to promote childcare services were not effective, owing to weak collaboration between childcare centers and local public sectors, off-target communication themes, and inefficient communication channels.

**What are the implications of the main findings?**

- Governments should strengthen communication of childcare services to target parents through dedicated attention and funding.
- Regular promotion of childcare services should be conducted based on a mode of public–private partnership and more efficient channels should be employed, especially new social media.

**Abstract:**

**Background/Objectives**: In China, developing childcare services is a key governmental strategy to promote child health under the low-fertility context. However, young couples have poor knowledge and low acceptance of formal childcare now, creating a significant gap between need and actual choice. To fulfil the unmet need and promote the development of childcare services, raising awareness among prospective parents is necessary. Guided by the theory of planned behavior and the theory of cultural transmission and evolution, this study evaluates whether current communication practices address the theoretically important factors pertaining to effective promotion. **Methods**: Taking provincial capital cities and sub-provincial cities as the study sample, we conducted a content analysis of policy documents related to communication of formal childcare and a social network analysis of practical promotional activities, respectively. **Results**: There were a series of problems with childcare service communication. First, governmental sectors failed to pay sufficient attention to communication of childcare services, and promotional activities were basically conducted as a campaign rather than a regular style. Second, there was little effective partnership between childcare centers and community committees. Third, promotion was mainly conducted through traditional channels, and new social media were less used. **Conclusions**: To improve the communication of childcare services to target parents, we recommend that governments (1) strengthen communication efforts through dedicated attention and funding; (2) establish regular communication based on public–private partnership modes; and (3) employ more efficient channels, especially social media.

## 1. Introduction

Child health is a foundational component of public health systems and childcare, which promotes children’s health and healthy habits (e.g., handwashing after meals and toileting) through early childhood care and education, is receiving attention again in China, and promoting the development of childcare services by various supports has been set as a priority strategy in the Healthy China Initiative [1]. The reason is clear. China is facing the challenge of very low fertility, and childcare services are expected to make multi-faceted contributions: promoting the health, growth, and development of children [2,3,4,5], alleviating work–family conflicts [6,7,8,9], and, thus, partly improving young couples’ intention to have a(nother) child and population fertility rates [10,11,12,13,14,15,16,17,18]. Since 2019, China has restarted the promotion of formal childcare services after 30 years of decline [19,20,21,22,23,24] and in the year, the State Council of the People’s Republic of China released “The Guiding Opinions on Promoting the Development of Care Services for Children Under 3 Years of Age”, which was widely regarded as a landmark in the revival of formal childcare services [19].

The theory of cultural transmission and evolution predicts that new norms spread slowly at the early stage of their transmission [25], which is exactly true with the case of the ideology of using formal childcare services among current Chinese parents. According to the State Council’s Report on Promoting the Development of Childcare Services, released in September 2024, more than 30% of couples with children under three years of age had a need for sending their children to childcare centers; however, the actual rate of enrolment into centers among 0–3-year-old children was just 7.86% [26]. The above national statistics were supported by ones at the provincial level. At the end of 2023, among 1,262,400 children under three years of age in Hunan Province, only 97,600 were enrolled in a childcare center, i.e., a rate of only 7.73% [27]. Similarly, only 9.5% of 1.29 million 0–3-year-old children in Zhejiang Province in 2022 were cared for in childcare centers [28]. Up to August 2023, the average number of childcare slots per 1000 persons in Beijing city was just 1.32; however, even under such a shortage of childcare space supply, the unused capacity ratio of registered childcare slots was higher than 70% [29]. Evidently, the center-based childcare rate in China is far lower than that in OECD countries, which stands at around 36% [30]. Further analysis shows that one of major reasons for the low attendance rate among such children is information asymmetry: parents of such children have no sufficient knowledge about formal childcare services; consequently, formal childcare has not become a customary choice [31,32,33]. Given the above facts, communicating the functions and merits of formal childcare services to prospective parents is warranted so that more parents with 0–3-year-old children will recognize and accept such a choice [34,35].

Given such a necessity, three factors should receive sufficient attention in communication of formal childcare services in current China. First, the theory of planned behavior predicts that those who benefit most from developing formal childcare services in China, especially governments planning to promote the provision of quasi-public childcare services and owners of childcare centers, will have the strongest motivation to take part in communication activities [36]. For instance, Wang and Liu (2022) [35] found that these owners dearly hoped that local governments, including quasi-public community committees overseen by social workers, could help them in distributing communication materials like leaflets. Second, the theory also predicts that effort should be taken to relieve negative beliefs towards center-based childcare, as favorable or unfavorable attitudes towards performing a given behavior determine directly intention to perform it; however, underlying such attitudes are beliefs that link the behavior with favorable or unfavorable outcomes [36]. For instance, the analysis of a sample of US women by Erdwins and Buffardi (1994) [37] suggested that positive beliefs towards exposure of children to other adults and children in daycare centers might explain maternal choice of center-based childcare services. Based on a latent class analysis of another US sample, Kim and Fram (2009) [38] showed that parents who emphasized learning of children were more likely to choose center-based care. Third, the theory of cultural transmission and evolution predicts that horizontal transmission, e.g., exchange of information between peers of similar ages through new social media like WeChat or TikTok in China, might be the most efficient approach in transmitting, in a changing social context, new norms, like utilizing formal childcare services that were not popular previously [25,39,40].

**Figure 1:**
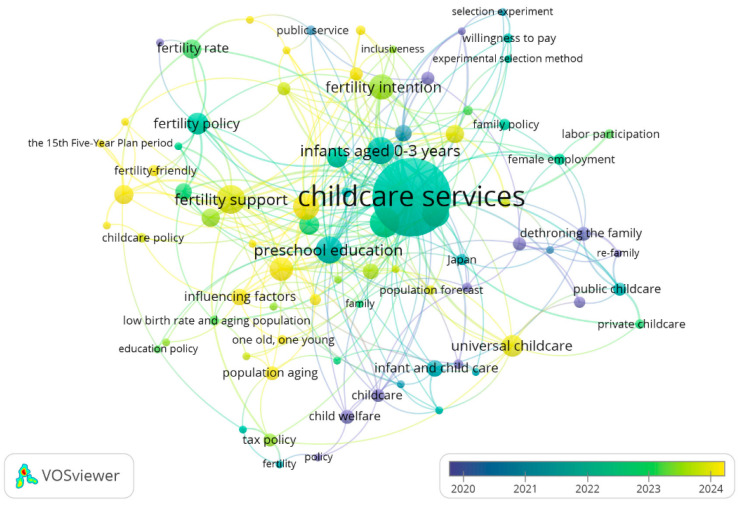
The co-occurrence of keywords in 422 childcare research articles published in core or CSSCI journals. One solid circle represents a theme/keyword, and the connection between the two circles means that the two themes appeared together in an article.

**Figure 2:**
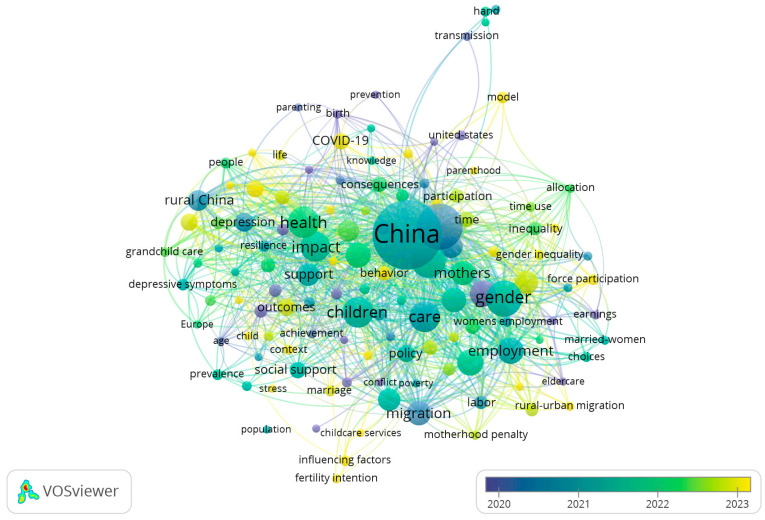
The co-occurrence of keywords in 357 research articles on childcare in China that were published in SCI or SSCI journals. One solid circle represents a theme/keyword, and the connection between the two circles means that the two themes appeared together in an article.

Up to now, there have been few studies that systematically evaluate the current status of communication of formal childcare services across China. More specifically, using “childcare” in Chinese (“tuoyu”) as the theme, a search of research articles indexed in CNKI, the most comprehensive and authoritative database of the academic literature published in Chinese, led to a total of 422 articles published in core or CSSCI journals (note: the search was conducted on 16 January 2026). Additionally, using “childcare” and “China” in English as the topics, a search of research articles indexed in the Web of Science (WOS) Core Collection led to a total of 357 articles published in SCI or SSCI journals (note: the search period was from 1 January 2006, to 16 January 2026). Based on co-occurrence analysis using VOSviewer (version 1.6.20), we found that these articles retrieved from either CNKI or WOS mainly focused on childcare services [8,12], childcare policies [41,42], fertility support [43], infants and toddlers aged 0–3 years [19,21], preschool education [33], (child’s) health [44], women’s employment [9], etc. (Figure 1 and Figure 2). In contrast, few articles revolved around the themes of “communication of childcare” or “communication of childcare services”.

To evaluate whether current childcare communication practices in China address the theoretically important factors identified above, this study tries to answer the following questions based on analyses of childcare policies and practical promotional activities, i.e., both policy design and implementation aspects. (1) Does the communication issue receive sufficient attention in governmental policies related to childcare? (2) Are the major stakeholders of childcare services, as predicted by the theory of planned behavior, involved in communication practice? Do they collaborate well in communication? (3) What themes are emphasized in communication practice? Do they target the beliefs shaping attitudes toward center-based childcare? (4) What channels are used, and do they facilitate horizontal transmission, as predicted by the theory of cultural transmission and evolution? Based on analysis results, we propose some policy implications for improving childcare service communication effectiveness in the context of low fertility.

## 2. Materials and Methods

**Figure 3:**
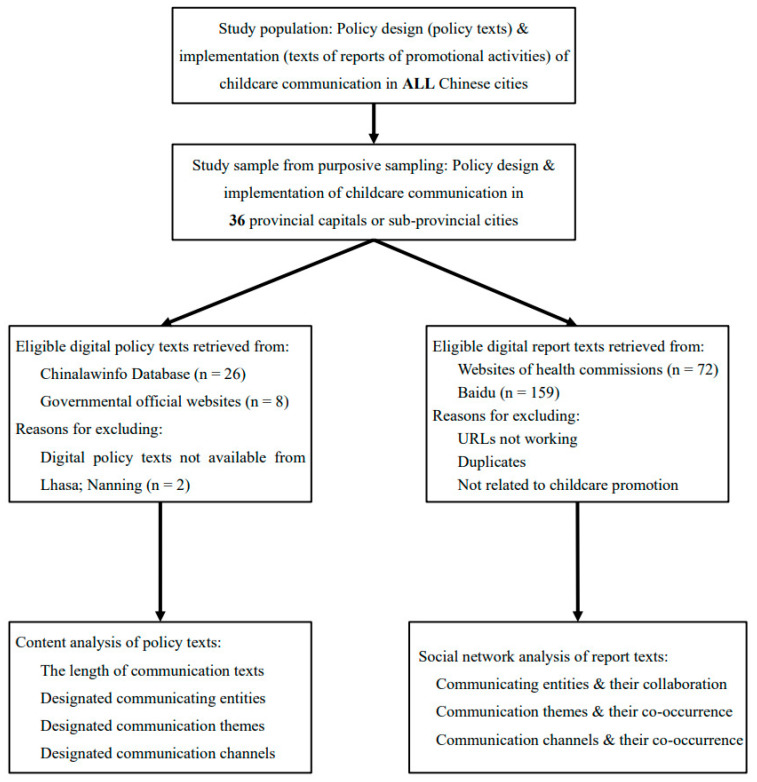
The design of the study population, sampling, data collection, and analyses.

The design of the research is summarized in Figure 3.

### 2.1. The Sample of Analyzed Cities

The population of analysis comprises all cities administered by a province or a similar unit, the first administrative level in China (population size ≈ 330 cities).

Using a purposive sampling strategy, we selected all 36 provincial capitals and sub-provincial cities, which hold similar status as provincial capitals, as the sample of analysis. First, from the perspective of representativeness, there is evident policy homogeneity within a province, and thus the childcare policy text of a selected city closely follows the corresponding provincial guideline and is similar to those of other cities in the same province. Second, from the perspective of data accessibility, such a city’s administration is generally more standardized due to its benchmark status within the province, e-government is more developed, and, consequently, texts on childcare policies and promotional reports are easier to collect than in other cities in the same province. In brief, the selection of these 36 cities as the analysis sample helps to collect two kinds of materials, as required for answering the above research questions.

### 2.2. Two Types of Textual Data: Childcare Policies and Reports of Promotional Activities

The first type of data used for analysis was policy texts. Since the State Council issued its landmark guidelines on early childhood care in 2019 (Supplementary File S1: PT000000), all 31 provinces or equivalent administrative units and cities under their administration released their own guiding policies; as mentioned above, the policy text of a given unit generally follows that of the adjacent higher-level government. We collected digital policy texts from official government websites or the Laws and Regulations Database (Chinalawinfo) [45]. A total of 34 texts were obtained: 8 from government websites and 26 from the Chinalawinfo database, covering 29 provincial capital cities (the policy texts of Lhasa and Nanning were not found online) and all 5 non-capital sub-provincial cities. These policy texts were then analyzed for their attention to communication of childcare services and arrangement of communication entities, themes, and channels. The full list of collected policy texts with URLs is shown in Supplementary File S1.

The other kind of data used for analysis was digital texts of news reports on communication about formal childcare services in practice. A report was extracted and included for analysis if (1) it came from the 36 sampled cities; (2) its content substantially involved communication of childcare services to target parents; (3) it was unique (i.e., excluding duplicates and retaining only the most complete version); and (4) its full text was available or the URL still worked. We collected digital report texts from two types of sources: government channels, especially the official websites of municipal health commissions (the regulatory bodies overseeing childcare centers); and Baidu, the most frequently used search engine in China. In each search, we used “childcare communication” or “childcare service communication” in Chinese as the search keywords. For government channels, all available reports on municipal health commission websites were reviewed. For Baidu, the first 20 pages of search results for each city were screened. Each report was then reviewed in full, and only those meeting all five inclusion criteria were retained for final analysis. A total of 231 news reports up to 31 December 2024 were collected from 29 provincial capital cities and four sub-provincial cities; among them, 72 were from government channels and 159 from Baidu (Note: no effective reports were collected from Shanghai, Lhasa, or Dalian). Due to the large number of cases, the complete list of all included reports with their titles and URLs is provided in Supplementary File S2.

### 2.3. Methods

#### 2.3.1. Content Analysis of Texts of Childcare Policies

For each policy document, we read the full text and identified all sentences and paragraphs concerning the communication of childcare-related matters.

Here is a typical example: “Popularize scientific child-rearing knowledge. Scientific parenting knowledge shall be disseminated through various channels including radio, television, community bulletin boards, and internet-based information twittering. Provide services including guidance on infant and toddler healthcare, safety protection, caregiving skills, and early childhood development to families and thereby, enhance their capacity for scientific child-rearing, by full use of resources from maternal and child health institutions, community health service centers (stations), infant and toddler care facilities, and childcare centers and through methods such as home visits, parent-child activities, parenting classes, and expert consultations. Each sub-district office (township/town government) should organize at least four parenting knowledge outreach activities annually. (Responsible Units: Municipal Publicity Department, Municipal Committee of the Communist Youth League, Municipal Women’s Federation, Municipal Family Planning Association, Municipal Health Commission, District/County/City Governments).” (Supplementary File S1: PT230100).

After extracting the above words on childcare communication, we then calculated the ratio of the word count of this paragraph to the total word count of policy documents of the city, i.e., Harbin. This ratio served as an indicator of the level of policy attention devoted to communication; the underlying rationale is that a larger proportion of communication-related content reflects the greater importance the government places on communicating about childcare. Then, we identified the following content directly from the text: (a) the main communication entities involved (e.g., the Municipal Publicity Department, the Municipal Committee of the Communist Youth League, etc.); (b) the communication themes addressed (e.g., scientific child-rearing knowledge, scientific parenting knowledge, infant and toddler healthcare, etc.); and (c) the communication channels used (e.g., home visits, parent–child activities, parenting classes, expert consultations, etc.).

#### 2.3.2. Social Network Analysis (SNA) of Reports of Promotional Activities

Before SNA, the communication entities, themes, and channels were identified from the texts of reports, following the procedure illustrated above.

Then, SNA allowed us to visualize and quantify how different entities, themes, and channels co-occurred across the 231 news reports to discover the underlying structure of these communication elements. In other words, the SNA diagrams not only illustrate which communication elements are present (note: this is somewhat the function of the above simple content analysis) but also demonstrate how they interact with each other. Individual units were represented by nodes, concurrence relationships between them were represented by edges, and nodes and edges together formed a social network. In each network diagram, the width of an edge was depicted as proportional to the total number of concurrence cases of two nodes connected by the edge (based on the 231 reports of promotional activities); the size of a node was depicted as proportional to the total number of other nodes connected to it; and the average strength of connections between nodes was measured by graph density, ranging between 0 and 1. The software used to analyze the social network was Gephi [46].

It is worth mentioning that in principle, SNA can also be used for texts of childcare policies. However, not as the case of reports of promotional activities, SNA figures of communication entities, themes, and channels will not provide much quantitative information here, as there were only 34 policy texts.

The trustworthiness of content analysis and social network analysis can be largely assured by the following facts: the length of communication-related texts in policies was counted using Microsoft Word, and communication entities, themes, and channels in policies and reports were identified by the authors straightforwardly from the texts, i.e., using the original keywords present in the texts, as illustrated above. In some rare cases of unstandardized expressions in texts, the two authors coded the content separately and then came to a consensus. To support transferability, we attach the detailed texts analyzed in the two appendices for confirmation (i.e., Supplementary Files S1 and S2).

To summarize, this is a quantitative empirical study of texts of policies and reports of childcare communication in China.

## 3. Results

### 3.1. Analysis of Policy Texts

#### 3.1.1. Attention Paid to Communication of Formal Childcare Services in Policy Documents

**Figure 4:**
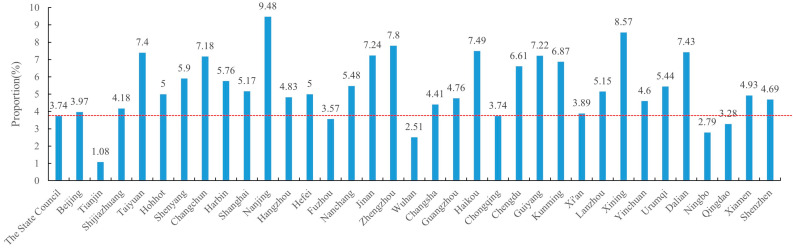
The ratio of text in the communication section to the total text volume in each policy document. The red dashed line indicates the level in the national document. The horizontal axis indicates where a policy document came from, and the vertical axis indicates the ratio value in the document.

Figure 4 illustrates the ratio of words in the communication section to the total text volume in each policy document released by The State Council, i.e., the national level, and 34 provincial capital cities or sub-provincial cities (Supplementary File S1) (note: communication was not limited to formal childcare services, as indicated below). The ratio was 3.74% at the national level. Most of the thirty-four analyzed cities displayed a ratio higher than this level, and the top three ratios were found in Nanjing (9.48%), Xining (8.57%), and Zhengzhou (7.8%); in contrast, Tianjin (1.08%) and Wuhan (2.51%) showed the lowest levels.

#### 3.1.2. The Designation of Communicating Entities in Policy Documents

Among the 34 cities studied, most of them designated communicating entities, but how they should be coordinated was not clearly arranged. The designated entities could include the Publicity Department, the Health Commission, the Federation of Trade Unions, the Communist Youth League, the Women’s Federation, the Family Planning Association, the Education Bureau, etc. In the long list, four departments, i.e., the Health Commission, the Family Planning Association, the Women’s Federation, and the Communist Youth League, stood out, and they were designated by at least 23 cities as the communicating entities. For instance, the document of Shijiazhuang city mentioned “Responsible departments: Municipal Health Commission, Municipal Women’s Federation, Municipal Youth League Committee, Municipal Education Bureau” (Supplementary File S1: PT130100). As a further instance, that of Xiamen city mentioned “Leading departments: Municipal Health Commission, Municipal Family Planning Association; responsible departments: Municipal Civil Affairs Bureau, Municipal Women’s Federation, Municipal Youth League” (Supplementary File S1: PT350200). Six cities did not designate governmental sectors responsible for policy communication of childcare services, including Beijing, Fuzhou, Guiyang, Ningbo, Changchun, and Zhengzhou.

It is worth noting that the designed communicating entities generally did not include quasi-public community committees, which are not formal administrative entities but typically staffed by non-governmental social workers. However, through implementation of government-contracted services, such committees function as administrative extensions. They are responsible for directly handling residents’ routine affairs, conveying notices or regulations from administrative entities and assisting them in managing community public affairs. Thus, grassroots social workers in China, while technically non-governmental, function as de facto local government agents.

#### 3.1.3. Recommended Themes of Communication in Policy Documents

There were two distinctive features in the themes of communication in these documents. First, although most of the cities under study included a heading for communication in their policy documents, almost all headings were level two ones, i.e., no documents had dedicated a level one heading for communication. Documents of seven cities did not even arrange a heading for communication: Guangzhou, Jinan, Lanzhou, Shijiazhuang, Tianjin, Changchun, and Hohhot. Second, although these documents were centered around “promoting the development of care services for children under 3 years of age”, few of them explicitly emphasized communication of formal childcare services (Supplementary File S1). Instead, so-called communication mentioned in these documents was mainly focused on guiding young parents to knowledge of science-based parenting and just touched on early care and education of 0–3-year-old children. For instance, the policy document of Chengdu city said “strengthen social communication and popularize science-based childcare knowledge…by leveraging maternal and child health institutions, community health service centers (stations), childcare centers, and early childhood development demonstration bases, deliver such services as infant healthcare, safety protection, caregiving skills, and early development guidance to families, through home visits, parent-child activities, parenting workshops, and internet-based platforms” (Supplementary File S1: PT510100).

#### 3.1.4. Recommended Channels of Communication in Policy Documents

Although a wide range of communication channels were mentioned by national and local policy documents, most of them belonged to traditional ones. Among them, parent–child activities, parenting workshops, and home-visiting guidance appeared in documents of at least 20 cities. For instance, the document of Lanzhou city said: “In accordance with parental demand for services, organize those activities suitable for physical and mental development of infants and toddlers and open a variety of science-based parenting lectures, parenting salons, expert consultations on a regular or non-regular basis, to enhance the parents’ knowledge of science-based parenting” (Supplementary File S1: PT620100). Similarly, the document of Ningbo city mentioned that “Provide specialized guidance on breastfeeding and science-based parenting for family-based childcare and thus, improve the proficiency of such childcare, through home-visiting guidance, parent-child activities, parenting lectures and twittering information of mother and child health handbook” (Supplementary File S1: PT330200). In contrast, only the document of Zhengzhou city explicitly mentioned some new social media: “Provide whole-processed, science-based and routine parenting guidance to local families with infants/toddlers and childcare centers and thus, effectively enhance their scientific childcare competencies, through WeChat official accounts, short videos and other information technologies as well as parent-child activities and parenting lectures” (Supplementary File S1: PT410100).

### 3.2. Analysis of Practice in Promoting Formal Childcare Services in Different Cities

In accordance with the “Notice on Launching the National Childcare Services Awareness Month” released by the National Health Commission, the analyzed activities of promoting childcare services were mainly held in a given month every year, generally in the style of a one-off push. In other words, such promotional activities were more of a perfunctory campaign than institutionalized practice, and thus their outreach was evidently inadequate.

#### 3.2.1. The Communicating Entities in Childcare Service Promotional Activities

**Figure 5:**
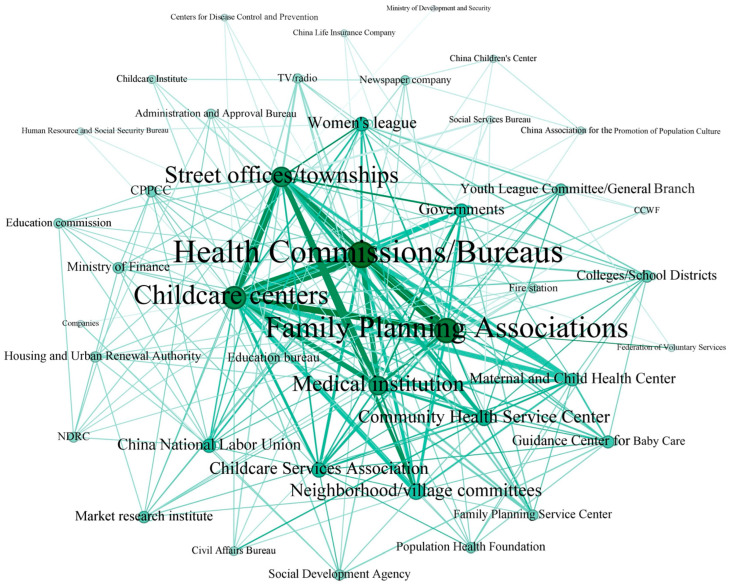
The network structure of communicating entities in reported activities for promoting formal childcare services. A node refers to an entity, and a line (i.e., an edge) connects it to another entity. The width of an edge is proportional to the total number of co-occurrences of two nodes (entities) in the 231 reported activities, and the size of a node is proportional to the total number of other nodes (entities) connected to it (CCWF: the China Children and Women’s Federation; CPPCC: the Chinese People’s Political Consultative Conference; NDRC: the National Development and Reform Commission).

The social network of communicating entities is shown in Figure 5. A node (solid circle) in the figure refers to one communicating entity, and an edge connects two entities who concurrently took part in one or more reported promotional activities. The network contains 41 nodes and 267 edges.

It can be seen that there were a wide range of entities involved in communication of childcare services (Figure 5). The three largest nodes in the network correspond to the Health Commissions/Bureaus (collaborating with a total of 36 other entities in 231 reported promotional activities), the Family Planning Associations (collaborating with a total of 32 other entities in 231 promotional activities), and childcare centers (collaborating with a total of 30 other entities in 231 promotional activities). At the same time, the edges connecting them are also relatively wider than the edges linking other entities, indicating that these three entities had been involved together in childcare promotional activities more frequently than other ones. In the 231 reported cases of promoting formal childcare, 75 of them mentioned collaboration between the Health Commissions/Bureau and childcare centers, 55 reports mentioned collaboration between the Health Commission/Bureau and the Family Planning Association, and 32 reports mentioned collaboration between the Family Planning Association and childcare centers.

Here, a concrete example is given to illustrate nodes and edges in the social network of communicating entities. In a promotional activity held in Wuhan on 3 July 2024, the news report mentioned: “In collaboration with the Maternal & Child Health Hospital of Qingshan District, the Health Bureau of Qingshan District organized a Childcare Awareness Month activity, which revolved around the theme of ‘Reliable Childcare, accessible and convenient’, at the Building of CDC and Maternal & Child Public Health of Qingshan District. Childcare centers and families with 0–3 year old children in the district participated in the activity. On the site, the Maternal & Child Hospital of the Qingshan District presented a popular lecture on infant and child healthcare, to guide parents to science-based childcare ideology and skills. Childcare centers communicated standards of childcare services, science-based childcare ideology and knowledge of early childhood care and education to parents” (Supplementary File S2: RT420100-10).

**Figure 6:**
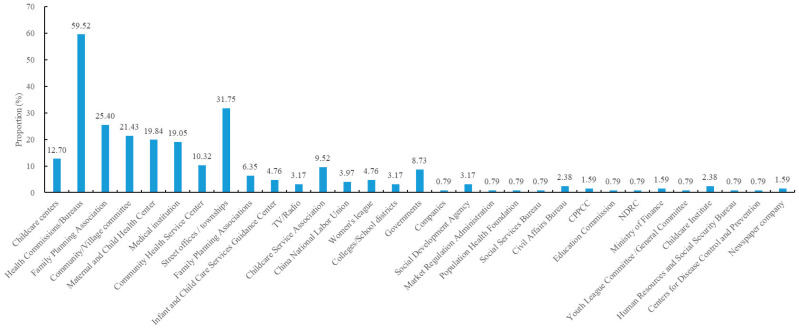
Collaborators with childcare centers in 126 reported promotional activities where at least one center was involved. The horizontal axis indicates collaborators, and the vertical axis indicates the likelihood that a given collaborator appeared in these activities (CPPCC: the Chinese People’s Political Consultative Conference; NDRC: the National Development and Reform Commission).

Thus, the public–private partnership mode for childcare service communication was preliminarily set up: there was relatively close collaboration between formal childcare centers and the governmental regulatory authority, i.e., the Health Commission, and related governmental supporting bodies like the Family Planning Association. However, there was no sign of close collaboration between childcare centers and community/village committees operated by social workers (Figure 5). Figure 6 further shows frequencies of collaboration between formal childcare centers and various entities in 126 reported promotional activities where at least one childcare center was involved. Evidently, only about 20% of cases involved a community/village committee. It is also worth mentioning that 12.7% of these activities were organized solely by childcare centers themselves.

#### 3.2.2. The Communication Themes in Childcare Service Promotional Activities

**Figure 7:**
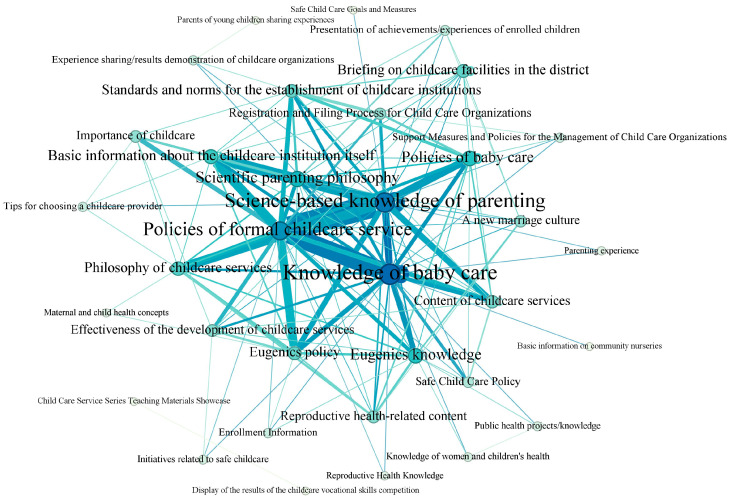
The network structure of communication themes in reported activities for promoting formal childcare services. A node refers to a theme, and a line (i.e., an edge) connects it to another theme. The width of an edge is proportional to the total number of co-occurrence of two nodes (themes) in the 231 reported activities, and the size of a node is proportional to the total number of other nodes (themes) connected to it.

Figure 7 shows the network of communication themes in childcare service promotional activities. There are 34 nodes in it, indicating an extensive range of communication themes. The four largest nodes correspond to science-based knowledge of parenting (25 other themes in total were communicated together with it in 231 reported promotional activities), knowledge of baby care (similarly, 25 other themes were communicated together with it), policies of formal childcare services (21 other themes were communicated together with it), and policies of baby care (14 other themes were communicated together with it). This indicates that childcare promotional campaigns were more focused on these aspects. Additionally, the edges between these nodes are wider than the ones between other nodes, i.e., the probabilities of their being communicated concurrently were higher. For instance, in 30 of 231 reported cases, science-based knowledge of parenting was communicated together with policies of formal childcare services; in 23 cases, baby care knowledge and policies of formal childcare services were communicated together. Evidently, thematic focus was basically consistent with the recommendation of policy texts, as mentioned above.

Here, a concrete example is given to illustrate nodes and edges in the social network of communication themes. In Shijiazhuang city, the Family Planning Association of the High-Tech District initiated a promotional activity on “Developing inclusive formal childcare services and promoting the implementation of the three-child policy” on 29 August 2023 (Supplementary File S2: RT130100-7). On the site, the personnel communicated the inclusive childcare policies to the public and disseminated knowledge about early childhood care and education for children under three years of age, e.g., growth and development, science-based parenting, health care, and safety and protection.

To summarize, the range of communication themes was broad, but they mainly focused on the ones recommended by policy documents. In contrast, those themes closely linked to selecting formal childcare services by reproductive-aged couples were more or less neglected, e.g., the importance of using formal childcare services before attending kindergarten, performance of children already in childcare centers, exhibition of service merits by childcare centers, and tips for choosing a qualified childcare center.

#### 3.2.3. The Communication Channels in Childcare Service Promotional Activities

**Figure 8:**
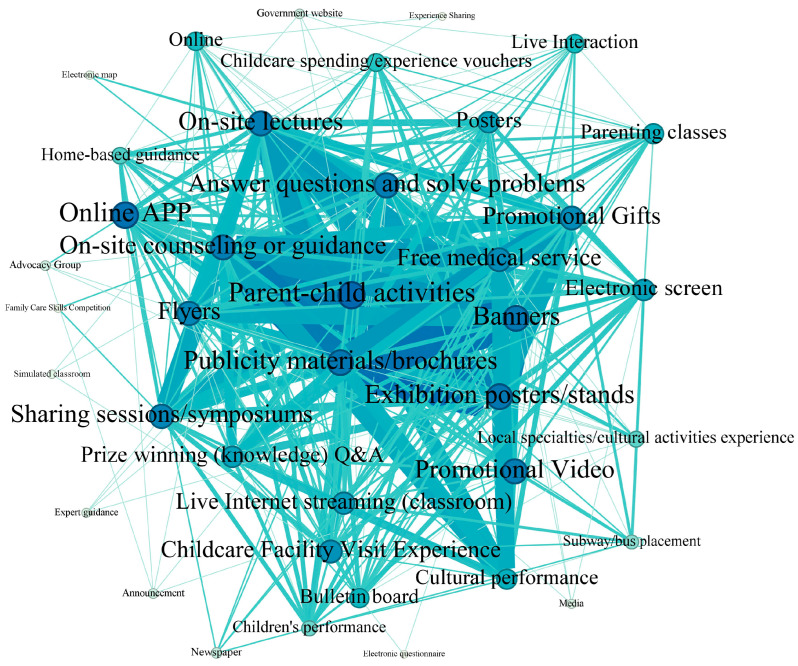
The network structure of communication channels in reported activities for promoting formal childcare services. A node refers to a channel, and a line (i.e., an edge) connects it to another channel. The width of an edge is proportional to the total number of co-occurrences of two nodes (channels) in the 231 reported activities, and the size of a node is proportional to the total number of other nodes (channels) connected to it.

Figure 8 illustrates the network of communication channels used in childcare service promotional activities. There are 39 nodes, most of which correspond to traditional channels of communication. The major channels, as indicated by larger nodes, were as follows: parent–child activities (used concurrently with 30 other channels in total in 231 reported activities), publicity materials, especially brochures (used concurrently with 28 other channels in total), hanging banners (used concurrently with 28 other channels in total), exhibition posters/stands (used concurrently with 28 other channels in total), on-site lectures (used concurrently with 27 other channels in total), and on-site counselling or guidance (used concurrently with 27 other channels in total). At the same time, the co-occurrence frequencies among these channels were also high, as illustrated by the wider edges connecting them. For instances, among the 231 reported promotional activities, publicity materials/brochures and posters/stands were employed in conjunction 65 times; publicity materials/brochures and banners were used together 59 times; on-site counselling and publicity materials/brochures were used together 53 times; and banners and posters/stands were used together 47 times.

Here, a concrete example is given to illustrate nodes and edges in the social network of communication channels. On 3 July 2024, Jinan City launched a series of promotional activities focusing on two themes: Childcare Service Promotion Month and healthy childbearing (Supplementary File S2: RT370100-9). The communication channels employed during these events included hanging banners, distributing publicity materials, cultural performances, on-site consultations, live presentations, parent–child activities, and free medical consultation stands.

Two features of the network illustrated in Figure 8 are worth mentioning. First, it has a graph density at 0.506 and a total of 375 edges. The density is larger than that of Figure 5 and Figure 7, i.e., the synergistic use of various communication channels was relatively more frequent than cases with communicating entities and themes. Second, largely consistent with the recommendation by policy documents, the channels shown in Figure 8 were predominantly offline ones, such as parent–child activities, publicity materials/brochures, exhibition posters/stands, and banners. Owing to their temporal and spatial limitations, such channels might not be sufficiently effective in attracting and serving the target group dearly in need of formal childcare services, i.e., reproductive-aged working couples, as these individuals have very limited opportunities to engage in on-site promotional activities held during weekdays. For instance, on a day in June 2023, The Guiyang Women’s Federation, in conjunction with the Yuhong Community of the Yuntan Subdistrict in the Guanshanhu District and Yuhong Parent School, organized a community public welfare lecture entitled “Inclusive childcare services, collective action” (Supplementary File S2: RT520100-1). During this event, the participants were predominantly middle-aged or elderly residents, whereas few young parents were present.

## 4. Discussion

As predicted by theories, there is strong demand for communication of formal childcare services to target audience in China currently, where such services are at an early stage of revival. Our systematic evaluation of the current status of communication indicates that such a need is clearly unmet, as reflected by both institutional neglect and weakness at the implementation level. Firstly, the guiding policies of both the central and local governments did not pay sufficient attention to the importance of communication in the development of formal childcare services. In practice, so-called promotional activities were conducted more or less as a one-off each year, rather than regularly. Secondly, although various communicating entities got involved in childcare service promotion, their collaboration relationship was not well arranged; in particular, the collaboration between quasi-public community committees operated by social workers and childcare centers was somewhat weak. Third, both policy documents and reported promotional activities mainly dealt with other themes than formal childcare services themselves; in particular, themes most related to young couples’ choice of services were rarely emphasized. Fourth, both policy documents and reported promotional activities mainly dealt with traditional channels, whereas those new social media, which are not restricted by temporal or spatial factors in efficiently transmitting ideology to targeted young couples, did not receive sufficient attention. The above findings could have some implications for promoting childcare services that are aimed at enhancing children’s health.

First, the theoretical argument and empirical analysis suggest that the communication challenge is not confined to China and might represent a typical phenomenon in the development of formal childcare services in the world. In-depth interviews with housewives with children under three years of age in South Korea by Kang and Lee (2016) [47] indicated that those interviewees thought that the country should strengthen communication of childcare services, as they did not know of such services or just learned of them through private channels like friends rather than governmental channels. Another study on childcare services in 2019–2023 South Korea by Kim (2024) [48] reported some problems in promoting childcare services similar to our findings. The channels used were primarily traditional ones, such as promotional souvenirs, flyers, posters, and banners; in contrast, contactless new media (including social network services, SNS) were little used, although their utilization has increased steadily since 2021. Work by Albanese and her collaborators brought to light some embarrassing problems in the development of formal childcare services in developed countries like Canada. The content analysis of articles on childcare services and policies in four influential Canadian newspapers from 2000 to 2007 indicated that there was little news coverage of these themes except for during election periods, i.e., 2005–2006; after that, coverage declined to its annual level in 2007 [49,50].

Second, our study also suggests some solutions for problems with communication of formal childcare services to target parents. Given that grassroots social workers (e.g., those in community/village-level committees) contact directly, frequently, and skillfully local residents [51], absorbing them into the communication entities, including also governmental regulatory authorities and supporting sectors and childcare centers, will lead to a more comprehensive and thus effective public–private partnership (PPP) for communication. On the other hand, the childcare centers themselves should shift their identity from service provider to advocate [52]. They are the true and key stakeholder of the childcare industry, and they need to communicate more actively formal childcare ideology to local residents. In practice, the governmental authorities regulating childcare services, i.e., the health commissions at various levels, can assign regular promotion tasks for both community committees and local childcare centers so that they need to collaborate closely to perform such duties. In collaborative promotion practice, some points are worth mentioning. One point is that the omitted communication themes mentioned above, e.g., performance of center-cared children and practical tips for choosing a qualified childcare center, should be integrated in communication. Next, communication channels should be more diverse. More specifically, besides traditional channels like public lectures, more efficient new social media popular among young parents, e.g., WeChat Official Account, TikTok, Kwai, and Rednote, should be actively used to achieve a full-coverage communication effect [53]. Lastly, governmental regulatory authorities should arrange special funds for promotional activities.

## 5. Conclusions

This study systematically examines the current state of communication of formal childcare services in China. Our study indicates that although childcare communication is in clear demand in China currently, it is evidently under-developed both in policy design and implementation. Insufficient policy attention is paid to childcare communication, it is campaign-style rather than regular promotional activities, there is weak collaboration among communicating entities, there is non-targeting communication content, i.e., deviating from the theoretically important topics and core concerns of target groups, and there is insufficient employment of new social media. Based on these findings, we propose the following recommendations to improve the effectiveness of childcare communication in China. First, policymakers and related governmental sectors should integrate communication work into the regular administration and budgeting of childcare services. Second, a collaboration mechanism to promote childcare services should be established so that there will be effective and synergistic collaboration between governmental sectors (certainly including grassroots community committees) and childcare centers. Third, communication content should focus on the practical concerns of young parents (benefits of childcare services for both children and working mothers, cost, safety, curriculum, etc.) rather than generic policy interpretation. Fourth, new social media platforms should be fully utilized, while traditional channels should be retained to reach groups with different information reception habits. These measures are expected to bridge the “last mile” between policy and target families, ultimately making formal childcare services more accessible for the intended population.

Despite the above findings, this study has also some limitations. First, our analysis was limited to policy documents and reports of promotional activities but did not incorporate direct feedback from families. Future research could employ surveys or interviews with grassroots social workers, childcare centers, and families to better understand challenges in communication practice. These challenges, together with other factors like socio-economic inequalities in access to childcare services, might explain low intention to utilize formal childcare services among targeted parents. Second, the countermeasures proposed in this study remain to be tested empirically. Future research could employ pilot interventions or experimental designs to validate the effectiveness of different communication strategies.

## Data Availability

The original contributions presented in this study are included in the article/Supplementary Materials. Further inquiries can be directed to the corresponding author.

## Supplementary Materials

The following supporting information can be downloaded at https://www.mdpi.com/article/10.3390/healthcare14060776/s1, File S1: URLs of policy texts of 34 cities analyzed; File S2: URLs of reported activities of 33 cities analyzed.
