# Supplementary material for: The Communication Issue in Developing Childcare Services for Children Under Three Years of Age in China: An Analysis of Policy Texts and Practical Cases
Source: Healthcare (Basel). 2026 Mar 19;14(6):776. doi: 10.3390/healthcare14060776 (PMC13027007; doi:10.3390/healthcare14060776)
Supplement: Supplementary file 1 [file healthcare-14-00776-s001.zip › healthcare-4150004-supplementary.pdf]

Supplementary File S1:

URLs of Policy Texts of 34 Cities Analysed

PT000000. State Council of the People's Republic of China: *The State council general office's guiding opinions on promoting the development of childcare services for children under 3 years of age*

https://www.pkulaw.com/chl/434b24b8b5769383bdfb.html?keyword=%E5%9B%BD%E5%8A%A1%E9%99%A2%E5%8A%9E%E5%85%AC%E5%8E%85%E5%85%B3%E4%BA%8E%E4%BF%83%E8%BF%9B3%E5%B2%81%E4%BB%A5%E4%B8%8B%E5%A9%B4%E5%B9%BC%E5%84%BF%E7%85%A7%E6%8A%A4%E6%9C%8D%E5%8A%A1%E5%8F%91%E5%B1%95%E7%9A%84%E6%8C%87%E5%AF%BC%E6%84%8F%E8%A7%81&way=listView

PT110000. Beijing, the national capital: *Opinions of the general office of the People's Government of Beijing Municipality on promoting the development of childcare services for children under 3 years of age*

https://www.pkulaw.com/lar/38571d2d3d58e02431e0a74593edc403bdfb.html?keyword=%E5%8C%97%E4%BA%AC%E5%B8%82%E4%BA%BA%E6%B0%91%E6%94%BF%E5%BA%9C%E5%8A%9E%E5%85%AC%E5%8E%85%E5%85%B3%E4%BA%8E%E4%BF%83%E8%BF%9B3%E5%B2%81%E4%BB%A5%E4%B8%8B%E5%A9%B4%E5%B9%BC%E5%84%BF%E7%85%A7%E6%8A%A4%E6%9C%8D%E5%8A%A1%E5%8F%91%E5%B1%95%E7%9A%84%E5%AE%9E%E6%96%BD%E6%84%8F%E8%A7%81&way=listView

(Note: the number in “PT110000” is the real administrative No. of Beijing city in China. The policy documents in other cities were numbered in the same way.)

PT120000. Tianjin city: *Notice of the general office of the Tianjin Municipal People's Government on issuing the implementation rules for promoting the development of childcare services for children under 3 years of age in Tianjin Municipality*

https://www.pkulaw.com/lar/5b1ff05b3621f327aea75495f402d545bdfb.html?keyword=%E5%A4%A9%E6%B4%A5%E5%B8%82%E4%BA%BA%E6%B0%91%E6%94%BF%E5%BA%9C%E5%8A%9E%E5%85%AC%E5%8E%85%E5%85%B3%E4%BA%8E%E5%8D%B0%E5%8F%91%E5%A4%A9%E6%B4%A5%E5%B8%82%E4%BF%83%E8%BF%9B3%E5%B2%81%E4%BB%A5%E4%B8%8B%E5%A9%B4%E5%B9%BC%E5%84%BF%E7%85%A7%E6%8A%A4%E6%9C%8D%E5%8A%A1%E5%8F%91%E5%B1%95%E5%AE%9E%E6%96%BD%E7%BB%86%E5%88%99%E7%9A%84%E9%80%9A%E7%9F%A5&way=listView

PT130100. Shijiazhuang, the capital of Hebei Province: *Notice of the general office of the Shijiazhuang Municipal People's Government on issuing the "key tasks for further deepening the medical and health system reform in Shijiazhuang Municipality", the "implementation*

opinions on reforming and improving the comprehensive supervision system for the medical and health industry" and the "implementation opinions on promoting the development of childcare services for children under 3 years of age"

https://www.sjz.gov.cn/columns/0c7e8c2d-93de-4591-bfea-dd4c962eaa0f/202003/27/8fefd860-ee07-44fb-a875-358ea0dfe16f.html

PT140100. Taiyuan, the capital of Shanxi Province: *Opinions of the general office of the Taiyuan Municipal People's Government on promoting the development of childcare services for children under 3 years of age*

https://www.pkulaw.com/lar/e39ff1770bf40d492bc687673a62648ebdfb.html?keyword=%E5%A4%AA%E5%8E%9F%E5%B8%82%E4%BA%BA%E6%B0%91%E6%94%BF%E5%BA%9C%E5%8A%9E%E5%85%AC%E5%AE%A4%E5%85%B3%E4%BA%8E%E4%BF%83%E8%BF%9B3%E5%B2%81%E4%BB%A5%E4%B8%8B%E5%A9%B4%E5%B9%BC%E5%84%BF%E7%85%A7%E6%8A%A4%E6%9C%8D%E5%8A%A1%E5%8F%91%E5%B1%95%E7%9A%84%E5%AE%9E%E6%96%BD%E6%84%8F%E8%A7%81&way=listView

PT150100. Hohhot, the capital of Inner Mongolia Autonomous Region: *Notice of the general office of the Hohhot Municipal People's Government on issuing several measures for promoting the development of childcare services in Hohhot Municipality* https://www.pkulaw.com/lar/31a3ea7460a94c884f1c8f225584f8d2bdfb.html?keyword=%E5%91%BC%E5%92%8C%E6%B5%A9%E7%89%B9%E5%B8%82%E4%BA%BA%E6%B0%91%E6%94%BF%E5%BA%9C%E5%8A%9E%E5%85%AC%E5%AE%A4%20%E5%85%B3%E4%BA%8E%E5%8D%B0%E5%8F%91%E5%91%BC%E5%92%8C%E6%B5%A9%E7%89%B9%E5%B8%82%E5%85%B3%E4%BA%8E%E4%BF%83%E8%BF%9B%E5%A9%B4%E5%B9%BC%E5%84%BF%E7%85%A7%E6%8A%A4%E6%9C%8D%E5%8A%A1%E5%8F%91%E5%B1%95%E8%8B%A5%E5%B9%B2%E6%8E%AA%E6%96%BD%E7%9A%84%E9%80%9A%E7%9F%A5&way=listView

PT210100. Shenyang, the capital of Liaoning Province: *Notice of the general office of the Shenyang Municipal People's Government on issuing the implementation plan for promoting the development of childcare services for children under 3 years of age*

https://www.pkulaw.com/lar/d18096317752621cb8f165bc9ad03d82bdfb.html?keyword=%E6%B2%88%E9%98%B3%E5%B8%82%E4%BA%BA%E6%B0%91%E6%94%BF%E5%BA%9C%E5%8A%9E%E5%85%AC%E5%AE%A4%E5%85%B3%E4%BA%8E%E5%8D%B0%E5%8F%91%20%20%E3%80%8A%E4%BF%83%E8%BF%9B3%E5%B2%81%E4%BB%A5%E4%B8%8B%E5%A9%B4%E5%B9%BC%E5%84%BF%E7%85%A7%E6%8A%A4%E6%9C%8D%E5%8A%A1%E5%8F%91%E5%B1%95%E5%AE%9E%E6%96%BD%E6%96%B9%E6%A1%88%E3%80%8B%E7%9A%84%E9%80%9A%E7%9F%A5&way=listView

PT210200. Dalian, a sub-provincial city in Liaoning Province: *Opinions of the general office of the Dalian Municipal People's Government on promoting the development of childcare services for children under 3 years of age*

https://www.dl.gov.cn/art/2020/9/10/art_2345_233404.html

PT220100. Changchun, the capital of Jilin Province: *Opinions of the general office of the Changchun Municipal People's Government on promoting the development of childcare services for children under 3 years of age*

https://www.pkulaw.com/lar/d671ca499d61b193ddbf22fdfb772ca0bdfb.html?keyword=%E9%95%BF%E6%98%A5%E5%B8%82%E4%BA%BA%E6%B0%91%E6%94%BF%E5%BA%9C%E5%8A%9E%E5%85%AC%E5%8E%85%E5%85%B3%E4%BA%8E%E6%8E%A8%E8%BF%9B%203%E5%B2%81%E4%BB%A5%E4%B8%8B%E5%A9%B4%E5%B9%BC%E5%84%BF%E7%85%A7%E6%8A%A4%E6%9C%8D%E5%8A%A1%E5%8F%91%E5%B1%95%E7%9A%84%E5%AE%9E%E6%96%BD%E6%84%8F%E8%A7%81&way=listView

PT230100. Harbin, the capital of Heilongjiang Province: *Notice of the general office of the Harbin Municipal People's Government on issuing the implementation plan for promoting the development of childcare services for children under 3 years of age in Harbin Municipality*

https://www.harbin.gov.cn/haerbin/c104532/202201/c01_64854.shtml

PT310000. Shanghai city: *Notice of the Shanghai Municipal People's Government on issuing the guiding opinions on promoting and strengthening childcare services for children under 3 years of age in the city*

https://www.pkulaw.com/lar/ea0949f6759fd41901debb4accec8f14bdfb.html?keyword=%E4%B8%8A%E6%B5%B7%E5%B8%82%E4%BA%BA%E6%B0%91%E6%94%BF%E5%BA%9C%E5%8D%B0%E5%8F%91%E3%80%8A%E5%85%B3%E4%BA%8E%E4%BF%83%E8%BF%9B%E5%92%8C%E5%8A%A0%E5%BC%BA%E6%9C%AC%E5%B8%823%E5%B2%81%E4%BB%A5%E4%B8%8B%E5%B9%BC%E5%84%BF%E6%89%98%E8%82%B2%E6%9C%8D%E5%8A%A1%E5%B7%A5%E4%BD%9C%E7%9A%84%E6%8C%87%E5%AF%BC%E6%84%8F%E8%A7%81%E3%80%8B%E7%9A%84%E9%80%9A%E7%9F%A5&way=listView

PT320100. Nanjing, the capital of Jiangsu Province: *Notice of the general office of the Nanjing Municipal People's Government on issuing the implementation plan for promoting the development of childcare services for children under 3 years of age in Nanjing Municipality*

https://www.pkulaw.com/lar/17af237636049df62edd6947723bdebabdfb.html?keyword=%E5%8D%97%E4%BA%AC%E5%B8%82%E4%BF%83%E8%BF%9B3%E5%B2%81%E4%BB%A5%E4%B8%8B%E5%A9%B4%E5%B9%BC%E5%84%BF%E7%85%A7%E6%8A%A4%E6%9C%8D%E5%8A%A1%E5%8F%91%E5%B1%95%E5%AE%9E%E6%96%BD%E6%96%B9%E6%A1%88%E7%9A%84%E9%80%9A%E7%9F%A5&way=listView

PT330100. Hangzhou, the capital of Zhejiang Province: *Notice of the general office of the Hangzhou Municipal People's Government on promoting the healthy development of childcare services for children under 3 years of age*

https://www.pkulaw.com/lar/d4104335a332ac16199b39baaf19679fbdfb.html?keyword=%E6%9D%AD%E5%B7%9E%E5%B8%82%E4%BA%BA%E6%B0%91%E6%94%BF%E5%BA%9C%E5%8A%9E%E5%85%AC%E5%8E%85%E5%85%B3%E4%BA%8E%E4%BF%83%E8%BF%9B3%E5%B2%81%E4%BB%A5%E4%B8%8B%E5%A9%B4%E5%B9%BC%E5%84%BF%E7%85%A7%E6%8A%A4%E6%9C%8D%E5%8A%A1%E5%81%A5%E5%BA%B7%E5%8F%91%E5%B1%95%E7%9A%84%E9%80%9A%E7%9F%A5&way=listView

PT330200. Ningbo, a sub-provincial city in Zhejiang Province: *Opinions of the general office of the Ningbo Municipal People's Government on promoting the development of childcare services for children under 3 years of age*

https://www.pkulaw.com/lar/4c3e4bc9983c7ef009eb2136145764e7bdfb.html?keyword=%E5%AE%81%E6%B3%A2%E5%B8%82%E4%BA%BA%E6%B0%91%E6%94%BF%E5%BA%9C%E5%8A%9E%E5%85%AC%E5%8E%85%E5%85%B3%E4%BA%8E%E4%BF%83%E8%BF%9B3%E5%B2%81%E4%BB%A5%E4%B8%8B%E5%A9%B4%E5%B9%BC%E5%84%BF%E7%85%A7%E6%8A%A4%E6%9C%8D%E5%8A%A1%E5%8F%91%E5%B1%95%E7%9A%84%E5%AE%9E%E6%96%BD%E6%84%8F%E8%A7%81&way=listView

PT340100. Hefei, the capital of Anhui Province: *Notice of the general office of the Hefei Municipal People's Government on issuing the implementation opinions on promoting childcare services for children under 3 years of age in Hefei Municipality*

https://www.hefei.gov.cn/xxgk/szfgb/2019/dshihao/szfbgswj/102533009.html

PT350100. Fuzhou, the capital of Fujian Province: *Notice of the general office of the Gulou District People's Government of Fuzhou Municipality on issuing the "implementation opinions on promoting childcare services for children under 3 years of age"*

https://www.gl.gov.cn/xjwz/zfxxgk/qzbmptlj/qrmzfjzfgzbm/glqzfb/fdzdgknr/gwgw/202005/t20200518_3280636.htm

(Note: given that the policy document of a district is generally consistent with that of the city administrating it (see main text), we used the district’s document to represent the city’s document, as the latter was not found online.)

PT350200. Xiamen, a sub-provincial city in Fujian Province: *Notice of the general office of the Xiamen Municipal People's Government on issuing the implementation plan for promoting the development of childcare services for children under 3 years of age*

https://www.pkulaw.com/lar/a86959fcf83531d2446fc5db04eee7e3bdfb.html?keyword=%E5%8E%A6%E9%97%A8%E5%B8%82%E4%BA%BA%E6%B0%91%E6%94%BF%E5%BA%9C%E5%8A%9E%E5%85%AC%E5%8E%85%E5%85%B3%E4%BA%8E%E5%8D%B0%E5%8F%91%E4%BF%83%E8%BF%9B3%E5%B2%81%E4%BB%A5%E4%B8%8B%E5%A9%B4%E5%B9%BC%E5%84%BF%E7%85%A7%E6%8A%A4%E6%9C%8D%E5%8A%A1%E5%8F%91%E5%B1%95%E5%AE%9E%E6%96%BD%E6%96%B9%E6%A1%88%E7%9A%84%E9%80%9A%E7%9F%A5&way=listView

PT360100. Nanchang, the capital of Jiangxi Province: *Notice of the general office of the Nanchang Municipal People's Government on issuing the implementation plan for the construction of childcare service system for children under 3 years of age in Nanchang Municipality*

https://www.pkulaw.com/lar/f7dac28ab7840156079d0fd5c2b3c78ebdfb.html?keyword=%E5%8D%97%E6%98%8C%E5%B8%82%E4%BA%BA%E6%B0%91%E6%94%BF%E5%BA%9C%E5%8A%9E%E5%85%AC%E5%8E%85%E5%85%B3%E4%BA%8E%E5%8D%B0%E5%8F%91%E5%8D%97%E6%98%8C%E5%B8%823%E5%B2%81%E4%BB%A5%E4%B8%8B%E5%A9%B4%E5%B9%BC%E5%84%BF%E7%85%A7%E6%8A%A4%E6%9C%8D%E5%8A%A1%E4%BD%93%E7%B3%BB%E5%BB%BA%E8%AE%BE%E5%AE%9E%E6%96%BD%E6%96%B9%E6%A1%88%E7%9A%84%E9%80%9A%E7%9F%A5&way=listView

PT370100. Jinan, the capital of Shandong Province: *Opinions of the general office of the Jinan Municipal People's Government on promoting the development of childcare services for children under 3 years of age*

https://www.pkulaw.com/lar/2da92da242bd26b42c72055f3f7d3385bdfb.html?keyword=%E6%B5%8E%E5%8D%97%E5%B8%82%E4%BA%BA%E6%B0%91%E6%94%BF%E5%BA%9C%E5%8A%9E%E5%85%AC%E5%8E%85%E5%85%B3%E4%BA%8E%E4%BF%83%E8%BF%9B3%E5%B2%81%E4%BB%A5%E4%B8%8B%E5%A9%B4%E5%B9%BC%E5%84%BF%E7%85%A7%E6%8A%A4%E6%9C%8D%E5%8A%A1%E5%8F%91%E5%B1%95%E7%9A%84%E5%AE%9E%E6%96%BD%E6%84%8F%E8%A7%81&way=listView

PT370200. Qingdao, a sub-provincial city in Shandong Province: *Opinions of the general office of the Qingdao Municipal People's Government on promoting the development of childcare services for children under 3 years of age*

https://www.pkulaw.com/lar/3e12b94dec0e04707d769437e2cdf751bdfb.html?keyword=%E9%9D%92%E5%B2%9B%E5%B8%82%E4%BA%BA%E6%B0%91%E6%94%BF%E5%BA%9C%E5%8A%9E%E5%85%AC%E5%8E%85%E5%85%B3%E4%BA%8E%E4%BF%83%E8%BF%9B3%E5%B2%81%E4%BB%A5%E4%B8%8B%E5%A9%B4%E5%B9%BC%E5%84%BF%E7%85%A7%E6%8A%A4%E6%9C%8D%E5%8A%A1%E5%8F%91%E5%B1%95%E7%9A%84%E5%AE%9E%E6%96%BD%E6%84%8F%E8%A7%81&way=listView

PT410100. Zhengzhou, the capital of Henan Province: *Implementation opinions on promoting the development of childcare services for children under 3 years of age*

https://zzrs.zhengzhou.gov.cn/flfg/9299885.jhtml

PT420100. Wuhan, the capital of Hubei Province: *Notice of the general office of the Wuhan Municipal People's Government on strengthening childcare services for children under 3 years of age*

https://www.pkulaw.com/lar/f5aad82a61ecd439587a1f426aa3adcabdfb.html?keyword=%E6%AD%A6%E6%B1%89%E5%B8%82%E4%BA%BA%E6%B0%91%E6%94%BF%E5%B

A%9C%E5%8A%9E%E5%85%AC%E5%8E%85%E5%85%B3%E4%BA%8E%E5%8A%A0%E5%BC%BA3%E5%B2%81%E4%BB%A5%E4%B8%8B%E5%A9%B4%E5%B9%BC%E5%84%BF%E7%85%A7%E6%8A%A4%E6%9C%8D%E5%8A%A1%E5%B7%A5%E4%BD%9C%E7%9A%84%E9%80%9A%E7%9F%A5&way=listView

PT430100. Changsha, the capital of Hunan Province: *Opinions of the general office of the Changsha Municipal People's Government on promoting the development of childcare services for children under 3 years of age*

https://www.pkulaw.com/lar/f4ce4c59d4e525c6d0da4f33f19aa3ebbdfb.html?keyword=%E9%95%BF%E6%B2%99%E5%B8%82%E4%BA%BA%E6%B0%91%E6%94%BF%E5%BA%9C%E5%8A%9E%E5%85%AC%E5%8E%85%E5%85%B3%E4%BA%8E%E6%8E%A8%E8%BF%9B3%E5%B2%81%E4%BB%A5%E4%B8%8B%E5%A9%B4%E5%B9%BC%E5%84%BF%E7%85%A7%E6%8A%A4%E6%9C%8D%E5%8A%A1%E5%8F%91%E5%B1%95%E7%9A%84%E5%AE%9E%E6%96%BD%E6%84%8F%E8%A7%81&way=listView

PT440100. Guangzhou, the capital of Guangdong Province: *Notice of the general office of the Inter-ministerial Joint Conference on Childcare Services for Children Under 3 Years of Age in Guangzhou Municipality on issuing the "Implementation plan for promoting childcare services for children under 3 years of age in Guangzhou Municipality"*

https://www.pkulaw.com/lar/72a97b755c889b95c82a556c71528073bdfb.html?keyword=%E5%B9%BF%E5%B7%9E%E5%B8%823%E5%B2%81%E4%BB%A5%E4%B8%8B%E5%A9%B4%E5%B9%BC%E5%84%BF%E6%89%98%E8%82%B2%E6%9C%8D%E5%8A%A1%E5%B7%A5%E4%BD%9C%E8%81%94%E5%B8%AD%E4%BC%9A%E8%AE%AE%E5%8A%9E%E5%85%AC%E5%AE%A4%E5%85%B3%E4%BA%8E%E5%8D%B0%E5%8F%91%E3%80%8A%E5%B9%BF%E5%B7%9E%E5%B8%82%E6%8E%A8%E8%BF%9B3%E5%B2%81%E4%BB%A5%E4%B8%8B%E5%A9%B4%E5%B9%BC%E5%84%BF%20%E7%85%A7%E6%8A%A4%E6%9C%8D%E5%8A%A1%E5%B7%A5%E4%BD%9C%E5%AE%9E%E6%96%BD%E6%96%B9%E6%A1%88%E3%80%8B%E7%9A%84%E9%80%9A%E7%9F%A5&way=listView

PT440300. Shenzhen, a sub-provincial city in Guangdong Province: *Notice of the general office of the Shenzhen Municipal People's Government on issuing the implementation plan for promoting the development of childcare services for children under 3 years of age (2020-2025)*

https://www.pkulaw.com/lar/933f4ecefd5db82ece5aea849a2fae84bdfb.html?keyword=%E6%B7%B1%E5%9C%B3%E5%B8%82%E4%BA%BA%E6%B0%91%E6%94%BF%E5%BA%9C%E5%8A%9E%E5%85%AC%E5%8E%85%E5%85%B3%E4%BA%8E%E5%8D%B0%E5%8F%91%E4%BF%83%E8%BF%9B3%E5%B2%81%E4%BB%A5%E4%B8%8B%E5%A9%B4%E5%B9%BC%E5%84%BF%E7%85%A7%E6%8A%A4%E6%9C%8D%E5%8A%A1%E5%8F%91%E5%B1%95%E5%AE%9E%E6%96%BD%E6%96%B9%E6%A1%88&way=listView

PT460100. Haikou, the capital of Hainan Province: *Opinions of the general office of the Haikou Municipal People's Government on promoting the development of childcare services*

for children under 3 years of age

https://www.pkulaw.com/lar/4b0e15b403104822766c27e9ee6067a3bdfb.html?keyword=%E6%B5%B7%E5%8F%A3%E5%B8%82%E4%BA%BA%E6%B0%91%E6%94%BF%E5%BA%9C%E5%8A%9E%E5%85%AC%E5%AE%A4%E5%85%B3%E4%BA%8E%E4%BF%83%E8%BF%9B3%E5%B2%81%E4%BB%A5%E4%B8%8B%E5%A9%B4%E5%B9%BC%E5%84%BF%E7%85%A7%E6%8A%A4%E6%9C%8D%E5%8A%A1%E5%8F%91%E5%B1%95%E7%9A%84%E5%AE%9E%E6%96%BD%E6%84%8F%E8%A7%81&way=listView

PT500000. Chongqing city: *Opinions of the general office of the Chongqing Municipal People's Government on promoting the development of childcare services for children under 3 years of age*

https://www.cq.gov.cn/zwgk/zfxxgkml/szfwj/xzgfxwj/szfbgt/201912/t20191231_8837731.html

PT510100. Chengdu, the capital of Sichuan Province: *Opinions of the general office of the Chengdu Municipal People's Government on promoting the development of childcare services for children under 3 years of age*

https://www.pkulaw.com/lar/a41793d40eaee42aff08385f80bb6526bdfb.html?keyword=%E6%88%90%E9%83%BD%E5%B8%82%E4%BA%BA%E6%B0%91%E6%94%BF%E5%BA%9C%E5%8A%9E%E5%85%AC%E5%8E%85%E5%85%B3%E4%BA%8E%E4%BF%83%E8%BF%9B3%E5%B2%81%E4%BB%A5%E4%B8%8B%E5%A9%B4%E5%B9%BC%E5%84%BF%E7%85%A7%E6%8A%A4%E6%9C%8D%E5%8A%A1%E5%8F%91%E5%B1%95%E7%9A%84%E5%AE%9E%E6%96%BD%E6%84%8F%E8%A7%81&way=listView

PT520100. Guiyang, the capital of Guizhou Province: *Notice of the general office of the Nanming District People's Government on issuing the "implementation plan for promoting the development of childcare services for children under 3 years of age in Nanming District"*

https://www.nanming.gov.cn/zwgk/zfxxgk/fdzdgknr/zcwj/nff/202110/t20211026_71279161.html

(Note: given that the policy document of a district is generally consistent with that of the city administrating it (see main text: lines 111–112), we used the district's document to represent the city's document, as the latter was not found online.)

PT530100. Kunming, the capital of Yunnan Province: *Opinions of the general office of the Kunming Municipal People's Government on promoting the development of childcare services for children under 3 years of age*

https://www.pkulaw.com/lar/99218074177bc56b4a5b8f0139c1fb7cbdfb.html?keyword=%E6%98%86%E6%98%8E%E5%B8%82%E4%BA%BA%E6%B0%91%E6%94%BF%E5%BA%9C%E5%8A%9E%E5%85%AC%E5%AE%A4%E5%85%B3%E4%BA%8E%E4%BF%83%E8%BF%9B3%E5%B2%81%E4%BB%A5%E4%B8%8B%E5%A9%B4%E5%B9%BC%E5%84%BF%E7%85%A7%E6%8A%A4%E6%9C%8D%E5%8A%A1%E5%8F%91%E5

%B1%95%E7%9A%84%E5%AE%9E%E6%96%BD%E6%84%8F%E8%A7%81&way=listView

PT610100. Xi'an, the capital of Shaanxi Province: *Notice of the general office of the Xi'an Municipal People's Government on issuing the implementation plan for promoting childcare services for children under 3 years of age*

https://www.pkulaw.com/lar/39a84fe4920a46e91588fa55f676a5f1bdfb.html?keyword=%E8%A5%BF%E5%AE%89%E5%B8%82%E4%BA%BA%E6%B0%91%E6%94%BF%E5%BA%9C%E5%8A%9E%E5%85%AC%E5%8E%85%E5%85%B3%E4%BA%8E%E5%8D%B0%E5%8F%91%E6%8E%A8%E8%BF%9B3%E5%B2%81%E4%BB%A5%E4%B8%8B%E5%A9%B4%E5%B9%BC%E5%84%BF%E7%85%A7%E6%8A%A4%E6%9C%8D%E5%8A%A1%E5%B7%A5%E4%BD%9C%E5%AE%9E%E6%96%BD%E6%96%B9%E6%A1%88%E7%9A%84%E9%80%9A%E7%9F%A5&way=listView

PT620100. Lanzhou, the capital of Gansu Province: *Notice of the general office of the Lanzhou Municipal People's Government on issuing the implementation plan for promoting the development of childcare services for children under 3 years of age in Lanzhou Municipality*

https://www.pkulaw.com/lar/5e166d5ee286c6548b99364b12f2a831bdfb.html?keyword=%E5%85%B0%E5%B7%9E%E5%B8%82%E4%BA%BA%E6%B0%91%E6%94%BF%E5%BA%9C%E5%8A%9E%E5%85%AC%E5%AE%A4%20%E5%85%B3%E4%BA%8E%E5%8D%B0%E5%8F%91%E5%85%B0%E5%B7%9E%E5%B8%82%E4%BF%83%E8%BF%9B3%E5%B2%81%E4%BB%A5%E4%B8%8B%E5%A9%B4%E5%B9%BC%E5%84%BF%E7%85%A7%E6%8A%A4%E6%9C%8D%E5%8A%A1%E5%8F%91%E5%B1%95%E5%AE%9E%E6%96%BD%E6%96%B9%E6%A1%88%E7%9A%84%E9%80%9A%E7%9F%A5&way=listView

PT630100. Xining, the capital of Qinghai Province: *Notice of the general office of the Xining Municipal People's Government on issuing the "implementation plan for promoting the development of childcare services for children under 3 years of age in Xining Municipality"*

https://www.pkulaw.com/lar/358eb4023ad4026d04b795778e65a958bdfb.html?keyword=%E8%A5%BF%E5%AE%81%E5%B8%82%E4%BA%BA%E6%B0%91%E6%94%BF%E5%BA%9C%E5%8A%9E%E5%85%AC%E5%AE%A4%E5%85%B3%E4%BA%8E%E5%8D%B0%E5%8F%91%E3%80%8A%E8%A5%BF%E5%AE%81%E5%B8%82%E5%85%B3%E4%BA%8E%E4%BF%83%E8%BF%9B3%E5%B2%81%E4%BB%A5%E4%B8%8B%E5%A9%B4%E5%B9%BC%E5%84%BF%E7%85%A7%E6%8A%A4%E6%9C%8D%E5%8A%A1%E5%8F%91%E5%B1%95%E7%9A%84%E5%AE%9E%E6%96%BD%E6%96%B9%E6%A1%88%E3%80%8B%E7%9A%84%E9%80%9A%E7%9F%A5&way=listView

PT640100. Yinchuan, the capital of Ningxia Hui Autonomous Region: *Notice of the general office of the Yinchuan Municipal People's Government on issuing the implementation opinions on promoting the high-quality development of childcare services for children under 3 years of age in Yinchuan Municipality*

https://www.pkulaw.com/lar/022517366aa7cc6a72c76c4a8bbb8064bdfb.html?keyword=%E9%93%B6%E5%B7%9D%E5%B8%82%E4%BA%BA%E6%B0%91%E6%94%BF%E5%BA%9C%E5%8A%9E%E5%85%AC%E5%AE%A4%E5%85%B3%E4%BA%8E%E5%8D%B0%E5%8F%91%20%E9%93%B6%E5%B7%9D%E5%B8%82%E4%BF%83%E8%BF%9B3%E5%B2%81%E4%BB%A5%E4%B8%8B%E5%A9%B4%E5%B9%BC%E5%84%BF%E7%85%A7%E6%8A%A4%E6%9C%8D%E5%8A%A1%E9%AB%98%E8%B4%A8%E9%87%8F%E5%8F%91%E5%B1%95%E5%AE%9E%E6%96%BD%E6%84%8F%E8%A7%81%E7%9A%84%E9%80%9A%E7%9F%A5&way=listView

PT650100. Urumqi, the capital of Xinjiang Uygur Autonomous Region: *Notice of the Urumqi Municipal People's Government on issuing the implementation plan for promoting the development of childcare services for children under 3 years of age*

https://www.pkulaw.com/lar/6b95d077b3264c30ff062738c0123515bdfb.html?keyword=%E4%B9%8C%E9%B2%81%E6%9C%A8%E9%BD%90%E5%B8%82%E4%BA%BA%E6%B0%91%E6%94%BF%E5%BA%9C%E5%8D%B0%E5%8F%91%E5%85%B3%E4%BA%8E%E4%BF%83%E8%BF%9B3%E5%B2%81%E4%BB%A5%E4%B8%8B%E5%A9%B4%E5%B9%BC%E5%84%BF%E7%85%A7%E6%8A%A4%E6%9C%8D%E5%8A%A1%E5%8F%91%E5%B1%95%E7%9A%84%E5%AE%9E%E6%96%BD%E6%96%B9%E6%A1%88%E7%9A%84%E9%80%9A%E7%9F%A5&way=listView

Supplementary File S2:

URLs of Reported Promotional Activities in 33 Cities Analysed

RT110000. Beijing, the national capital (11 reported promotional activities in total)

(Note: the number in “RT110000” is the real administrative No. of Beijing city in China. The reported activities in other cities were numbered in the same way.)

RT110000-1:

Chaoyang district builds professionalized, standardized and high-quality childcare institutions to make parenting no longer difficult.

https://www.beijing.gov.cn/ywdt/gqrd/202312/t20231204_3489688.html

RT110000-2:

2023 "infant and toddler care week" thematic promotion campaign launches in Beijing.

https://baby.sina.com.cn/news/2023-06-02/doc-imyvwmrk8584708.shtml

RT110000-3:

Developing Shunyi childcare, benefiting thousands of households - Shunyi district childcare service promotion season officially begins.

https://www.sohu.com/a/799225556_121106842

RT110000-4 - RT110000-7:

Welcome to experience! Changping childcare promotion season series activities continue in progress. https://www.163.com/dy/article/JBM4PN6A0514DP1C.html

RT110000-8:

The city's first! Changping district childcare promotion volunteer service team is established. https://www.sohu.com/a/803462810_121106842

RT110000-9:

Fengguan road community carries out "reliable childcare, accessible and convenient" awareness month activities.

http://www.bjft.gov.cn/ftq/jxdt/202407/7b0e09e423b34fb182458b50b81ceb62.shtml

RT110000-10:

https://v.douyin.com/tNoLEYI4-9k/

RT110000-11:

https://v.douyin.com/l0GxZQlAkmc/

RT120000. Tianjin, city (3 reported promotional activities in total)

RT120000-1:

Nankai district carries out "inclusive childcare, joint action" childcare service awareness month activities.

https://mp.weixin.qq.com/s?__biz=MzA3MTMxMjk4NA==&mid=2247513933&idx=2&sn=4246709ea340401cb4702e61f692c485&chksm=9f2d847aa85a0d6c7297936de734879ea9930e17846a6d6e4835e868008e5b7007b91d2b8180&scene=27

RT120000-2:

Community helps you care for children free of charge, "child-friendly - baby house" public welfare activity is about to launch.

https://tj.news.163.com/23/0815/14/IC6HUQ0J042098PG.html

RT120000-3:

Tianjin: holds "reliable childcare, accessible and convenient" themed childcare service awareness month launch ceremony.

https://www.chinafpa.org.cn/xwzx/gdkx/202407/t20240701_18933.html

RT130100. Shijiazhuang, the capital of Hebei Province (7 reported promotional activities in total)

RT130100-1:

Yuhua district Health Bureau holds "inclusive childcare, joint action" infant and toddler care promotion campaign.

https://mp.weixin.qq.com/s?__biz=MzI3MjAwODg1MQ==&mid=2650886322&idx=5&sn=87f7bae263d288b4da9bb3b5ec5a2a43&chksm=f0cce34ec7bb6a585ce791b7e73391d86bf8a343beb5f42e7da13a16b5aeb686001411d5dff5&scene=27

RT130100-2:

Zanhuang county Health Bureau carries out "scientific parenting enters communities" series activities. https://news.sohu.com/a/721859046_121123711

RT130100-3:

"Inclusive childcare, Chang'an in action" Shijiazhuang Chang'a district holds childcare service awareness month launch ceremony.

https://shequ.hebnews.cn/2023-06/15/content_9020463.htm

RT130100-4:

http://www.yuhuaqu.gov.cn/col/1389844026937/2023/07/20/1689817889738.html

RT130100-5:

Childcare inclusivity, joint action - Yuhua fourth kindergarten childcare service awareness month activities.

https://mp.weixin.qq.com/s?__biz=MzIyNjg1MDAxNA==&mid=2247593650&idx=1&sn=09a9066b009c3b6578cf85179b2afe91&chksm=e8690b03df1e821555d49070e74846f0bf9d016a932817a9aa4df6d531a1a7cbbb6bad6b4725&scene=27

RT130100-6:

http://www.yuhuaqu.gov.cn/col/1389844026937/2023/07/20/1689818371046.html

RT130100-7:

Shijiazhuang high-tech district Family Planning Association conducts "developing inclusive childcare services, supporting three-child policy implementation" promotion campaign. https://sjz.hebnews.cn/2023-09/01/content_9061946.htm

RT140100. Taiyuan, the capital of Shanxi Province (3 reported promotional activities in total)

RT140100-1:

Shanxi provincial government Changzhi road kindergarten childcare center enters community to conduct "inclusive childcare, joint action" promotion activities.

http://www.sxjgsw.gov.cn/gzdt/sjdt/jsdw/art/2023/art_b0d90ccc584f4e4a9d9679816bca3282.html

RT140100-2:

Jinyuan district 2024 childcare service awareness month activities.

https://www.taiyuan.gov.cn/xqdt/20240729/30148874.html

RT140100-3:

Yingze district public comprehensive childcare service center officially opens.

http://sx.news.cn/20240712/cb40fdb91b3246e486a6a3e633d742bb/c.html

RT150100. Hohhot, the capital of Inner Mongolia Autonomous Region (1 reported promotional activity in total)

RT150100-1:

Inner Mongolia autonomous region Health Commission and Hohhot city jointly held the launch ceremony of regional childcare service awareness month and scientific parenting knowledge entering communities & households thematic campaign.

http://wjw.nmg.gov.cn/zwgk/xxgk/jtfz/202305/t20230531_2323973.html

RT210100. Shenyang, the capital of Liaoning Province (2 reported promotional activities in total)

RT210100-1:

Shenbei new district trade union conducts "inclusive childcare, joint action" childcare service thematic promotion week activities. https://news.sohu.com/a/694326674_121123868

RT210100-2:

Liaoning provincial Health Commission carries out "inclusive childcare, joint action" childcare service awareness month - entering community's activities.

https://baijiahao.baidu.com/s?id=1767766160720525423&wfr=spider&for=pc

RT220100. Changchun, the capital of Jilin Province (5 reported promotional activities in total)

RT220100-1:

"Inclusive childcare, health promotion, Jilin in action" provincial awareness month launch ceremony is held in Shuangyang district, Changchun city.

https://weibo.com/3523970940/N6bmQtd2Z

RT220100-2:

http://jl.zhonghongwang.com/show-216-28957-1.html

RT220100-3:

Reliable childcare, accessible and convenient - Changchun Jingyue high-tech zone Health Commission conducts childcare service promotion activities. https://i.ifeng.com/c/8c4M3c5vK72

RT220100-4:

Developing infant and toddler care services, promoting family harmony and happiness. https://mp.weixin.qq.com/s?__biz=MzU0MjAxMjg3Mw%3D%3D&mid=2247506482&idx=1&sn=f65b0a90c416173452429951bf39d56b&chksm=fad9f2311c40a202c61a7ed4909b1c51017c0299da4a896c5eefaceb791872fa81ab7d44f7e9&scene=27

RT220100-5:

"Spring babies" gathering women's strength, "supporting" new hopes for spring city. https://www.sohu.com/a/853505945_121106822

RT230100. Harbin, the capital of Heilongjiang Province (2 reported promotional activities in total)

RT230100-1:

Inclusive childcare, happy ten thousand households | Daoli district carries out childcare service promotion cultural performance.

https://mp.weixin.qq.com/s?__biz=MzI1MjgwOTQ3Ng%3D%3D&mid=2247720107&idx=3&sn=4595f72cbed54a57cbe2e3345ce19f30&chksm=e84beac61d536ddbfa612a88ddb6ee8ce618fc370aab64f357ad4ea6c8f5731f000a9672c981&scene=27

RT230100-2:

Harbin inclusive childcare service promotion and publicity effect is significant. https://baijiahao.baidu.com/s?id=1841612795140870989&wfr=spider&for=pc

RT320100. Nanjing, the capital of Jiangsu Province (5 reported promotional activities in total)

RT320100-1:

New district Yanjiang street carries out childcare service awareness month square activities. https://njna.nanjing.gov.cn/jd/yjjd/jddt/202306/t20230626_3946720.html

RT320100-2:

Jiangsu province and Nanjing city childcare service awareness month launches.

https://www.js.gov.cn/art/2024/6/16/art_60085_11271492.html

RT320100-3:

Jiangbei new district holds 2024 childcare service awareness month activities and "Xiaobei childcare" system launch ceremony.

https://njna.nanjing.gov.cn/xwzx/xqyw/202406/t20240627_4701204.html

RT320100-4:

https://www.yangtse.com/zncontent/3830746.html

RT320100-5:

Jiangsu Nanjing: 2024 Qinhuai district childcare service awareness month activities are held. https://www.nbs.cn/news/5/202407/t20240729_726555.html

RT330100. Hangzhou, the capital of Zhejiang Province (7 reported promotional activities in total)

RT330100-1:

Yiqiao town carries out childcare service policy promotion activities entering communities. https://www.sohu.com/a/740697087_121124214

RT330100-2:

Yiqiao town conducts reassuring childcare warming hearts promotion campaign.

https://roll.sohu.com/a/740911968_121124214

RT330100-3:

Zhejiang province holds 2024 childcare service awareness month activities and releases provincial childcare service logo. https://www.sohu.com/a/791895361_121118851

RT330100-4:

2024 Binjiang district childcare service awareness month campaign officially launches.

https://hznews.hangzhou.com.cn/chengshi/content/2024-05/13/content_8728077.htm

RT330100-5:

Hangzhou city carries out "eight ones" childcare service awareness month activities.

https://www.163.com/dy/article/J7VP853I0538AF52.html

RT330100-6:

Enjoying summer, "meeting" wonderful moments | parenting radio program enters communities - "Hang Xiaoyu" takes children garden touring.

https://mp.weixin.qq.com/s?__biz=MzA5ODIyODgzNw==&mid=2650550782&idx=1&sn=f21355a80ddc2dd440bfa9c119aa6fc3&chksm=89a5bbfc7418c49717b8bbb83267765651976eabaf69a308c5ec8793c602ccecb70db835f918&scene=27

RT330100-7:

"Hang Xiaoyu" arrives! Helping you avoid getting lost on the parenting journey.

https://act.thehour.cn/epaper/article/2024-07-10/4247975

RT330200. Ningbo, a sub-provincial city in Zhejiang Province (3 reported promotional activities in total)

RT330200-1:

Empowering "quality care for the young", Jiangbei district's 8th childcare awareness month arrives. https://www.nbjb.gov.cn/art/2023/11/23/art_1229105125_58954755.html

RT330200-2:

Over a hundred families participate in Haishu district childcare garden fair activities. https://www.haishu.gov.cn/art/2024/7/15/art_1229100495_58998061.html

RT330200-3:

Reliable childcare, accessible and convenient - district childcare service awareness month activities commence. https://www.zh.gov.cn/art/2024/7/31/art_1229724585_59329061.html

RT340100. Hefei, the capital of Anhui Province (29 reported promotional activities in total)

RT340100-1:

Inclusive childcare, we are in action - Feidong county Health Commission, Hefei city conducts reassuring childcare awareness month activities.

https://life.china.com/2023-06/29/content_221170.html

RT340100-2:

Feidong county future light preschool education group headquarters promotes "reassuring

childcare" campaign through multiple channels. https://ah.ifeng.com/c/8NYegBMye83

RT340100-3:

Tianle community carries out "reassuring childcare" warming hearts promotion campaign. https://www.hefei.gov.cn/zwgk/public/17431/109476791.html

RT340100-4:

https://www.linquan.gov.cn/xxgk/detail/628b442388668880448b4572.html

RT340100-5:

Hefei economic and technological development zone: launching policy outreach for childcare services to warm hearts.

http://edu.anhuinews.com/czsx/202211/t20221128_6534825.html

RT340100-6:

Reassuring childcare, warm extension - Hefei city Xueji road kindergarten promotes reassuring childcare campaign. https://www.ahyouth.com/news/20230828/1709814.shtml

RT340100-7:

Shuangdun town: childcare enters communities, services warm people's hearts.

https://www.hf365.com/2023/0417/1474302.shtml

RT340100-8:

Hefei Baohe district Ludu village: continuously promoting "reassuring childcare" warming hearts campaign. http://news.ahwang.cn/zhengwu/20230608/2525586.html

RT340100-9:

New station high-tech zone childcare-enterprise cooperation promotion activity launches in enterprises. https://www.hefei.gov.cn/zwgk/public/13801/109211249.html

RT340100-10:

Anhui province and Hefei city childcare service awareness month launch ceremony and "5.29" Family Planning Association member day event are held in new station high-tech zone.

https://wjw.ah.gov.cn/public/7001/56769111.html

RT340100-11:

Shuangdun town: community assistance, childcare for all.

https://www.hf365.com/2023/0608/1485705.shtml

RT340100-12:

Reassuring childcare, nurturing children's hearts.

https://www.hefei.gov.cn/ssxw/ztzl/zt/hfjkkxklxx/108918744.html

RT340100-13:

Changfeng (Shuangfeng) economic development zone carries out "developing childcare services, supporting three-child policy" volunteer promotion activities.

http://ah.anhuinews.com/hf/yq/202206/t20220613_6077559.html

RT340100-14:

Lantern festival service warms people's hearts - reassuring childcare, healthy growth.

http://ah.anhuinews.com/hf/news/xqjd/202302/t20230206_6657327.html

RT340100-15:

Reassuring childcare in action - Hefei Kaixuanmen kindergarten conducts warming hearts promotion. http://sqr.ahnews.com.cn/news/2023/08/30/c_407445.htm

RT340100-16:

Shushan district warming hearts reassuring childcare campaign fourth quarter work implementation situation. https://www.hefei.gov.cn/zwgk/public/13851/108554772.html

RT340100-17:

Hefei Yaohai district: "reassuring childcare" escorts growth and warms people's hearts. https://www.hfyaohai.gov.cn/yhzt/ztlb/msgc/mtjj/11324399.html

RT340100-18:

High-tech zone implements multiple measures to promote warming hearts reassuring childcare campaign. https://gxq.hefei.gov.cn/msgc/gzdt/18659971.html

RT340100-19:

Guanhu neighborhood committee: carries out "reassuring childcare protecting growth" childcare service promotion.

https://www.baohe.gov.cn/xxgk/zzjg/jzjyq/bhsjsq/gzdt/11303067.html

RT340100-20:

Baohe community Family Planning Association conducts childcare service promotion activities. https://www.baohe.gov.cn/xxgk/zzjg/jzjyq/bgjd/gzdt/11278107.html

RT340100-21:

Yaohai district Sanlijie street: Tielu yicun community carries out "reassuring childcare, warming hearts campaign" volunteer service activities.

http://hf.wenming.cn/zyfw/202303/t20230301_7984579.shtml

RT340100-22:

Qingfeng neighborhood committee: conducts "reassuring childcare" warming hearts campaign promotion activities.

https://www.baohe.gov.cn/xxgk/zzjg/jzjyq/bhsjsq/gzdt/11303881.html

RT340100-23:

Hefei city and economic development zone launch event for childcare services awareness month held.

http://ah.anhuinews.com/gdxw/202407/t20240707_7674678.html

RT340100-24:

"Peace of mind childcare, convenient and accessible" — Bantang sub-district launches 2024 "childcare awareness month" activities.

https://ahchjkq.hefei.gov.cn/ztzl/wmcs/18739645.html

RT340100-25:

Zhougudui community care for Next Generation Committee, Tong'an sub-district, Baohe district, Hefei city, in collaboration with international trade apartment Kindergarten, held the "reassuring, accessible childcare" awareness campaign.

http://www.china-torch.cn/txyd/jicengjujiao/2024-07-09/232943.html

RT340100-26:

"Hexin" co-parenting, entrusting the future - Hefei high-tech zone Xingyuan center conducts childcare service awareness month activities.

http://sqr.ahnews.com.cn/news/2024/07/16/c_555473.htm

RT340100-27:

Hefei Shushan economic and technological development zone childcare awareness month activity summary. http://sqr.ahnews.com.cn/news/2024/07/24/c_560230.htm

RT340100-28:

Hefei Haiheng Education Shimen road kindergarten: sending teaching into communities, childcare warming people's hearts.

https://so.html5.qq.com/page/real/search_news?docid=70000021_39465a2180c73852&faker=1

RT340100-29:

Luyang district Shuanggang street: inclusive childcare, joint action.

https://www.csjcs.com/news/show/28890e97aadda79e.html

RT350100. Fuzhou, the capital of Fujian Province (7 reported promotional activities in total)

RT350100-1:

Quality childcare, jointly nurturing the future | Kangaroo Kids "childcare service month" series activities review. https://news.sohu.com/a/694759475_121296921

RT350100-2:

Inclusive childcare, joint action - Jin'an district Health Commission conducts childcare service awareness month activities.

http://www.fzja.gov.cn/xjwz/zwgk/gzdt/bmdt/202305/t20230531_4612477.htm

RT350100-3:

Three lanes and seven alleys community: carries out childcare service awareness month campaign.

https://www.gl.gov.cn/xjwz/zwgkml/gzdt/sqdt/sqdt_sfqxsq/202306/t20230602_4614013.htm

RT350100-4:

Changle district launches childcare service awareness month activities.

http://www.fzcl.gov.cn/xjwz/wsbs/kstd/xh/202307/t20230705_4632309.htm

RT350100-5:

Inclusive childcare publicity teaches parents to take care of their children scientifically.

https://baijiahao.baidu.com/s?id=1767689439012200428&wfr=spider&for=pc

RT350100-6:

Guxi street: reliable childcare, accessible and convenient - Guxi street organizes childcare service awareness month thematic activities.

https://www.gl.gov.cn/xjwz/zwgkml/gzdt/bmdt_1/bmdt_gxjd/202407/t20240703_4853942.htm

RT350100-7:

Local Family Planning Associations carry out promotion campaigns to advance accessible childcare services. https://tv.fjsen.com/2024-08/12/content_31708691.htm

RT350200. Xiamen, a sub-provincial city in Fujian Province (14 reported promotional activities in total)

RT350200-1:

Fujian launches 2023 national childcare service awareness month campaign.

https://www.sohu.com/a/678374946_120578424

RT350200-2:

Living up to the "care" - Xiamen holds "neighborhood health" World Population Day and

infant & toddler care service thematic promotion event.

https://baijiahao.baidu.com/s?id=1770909559404744716&wfr=spider&for=pc

RT350200-3:

"Inclusive childcare, joint action!" - Siming district childcare service and World Population Day promotion activity is successfully held.

https://roll.sohu.com/a/694663727_121124352

RT350200-4:

Xiamen initiates "healthy Xiamen ·neighborhood health" World Population Day and infant & toddler care service thematic campaign.

https://hfpc.xm.gov.cn/xwzx/xwzx/202207/t20220712_2673926.htm

RT350200-5:

http://fj.zhonghongwang.com/show-201-29240-1.html

RT350200-6:

2024 Xiamen inclusive childcare service demonstration year launch ceremony is held in Jimei. https://xm.fjsen.com/wap/2024-07/14/content_31687483_0.htm

RT350200-7:

"I feel completely at ease leaving my baby with you!" Xiamen childcare services: policy support, association efforts, jointly solving parenting worries.

https://baijiahao.baidu.com/s?id=1820125975454769179&wfr=spider&for=pc

RT350200-8:

With dedication, warmth and assurance - families and childcare centers moving towards each other. https://cj.sina.com.cn/articles/view/2729129850/a2ab377a02001f5le

RT350200-9 - RT350200-14:

All districts of Xiamen carry out childcare policy and service knowledge promotion.

https://mp.weixin.qq.com/s?__biz=MzI3NTY3MjM1Mw==&mid=2247584338&idx=2&sn=f75fb5acb0f9b4a86815498fa40d3591&chksm=ea7df69a4e710b79e70f443ed914cfdf16d78abe4e8d4e0ef0ab42a97b04b438c0e2eb2d2702&scene=27

RT360100. Nanchang, the capital of Jiangxi Province (7 reported promotional activities in total)

RT360100-1:

Explore the childcare market: publicity across the country to help you understand childcare. https://www.sohu.com/a/706935870_121498866

RT360100-2:

Nanchang, Jiangxi: cohesion, creation of carriers, and excellent services to fully promote the construction of a new marriage and childbearing culture.

https://www.163.com/dy/article/IHRQQ2HH0514CJV0.html

RT360100-3:

Jiangxi Family Planning Association carried out the "5.29 Family Planning Association member activity day" publicity and service activities.

https://roll.sohu.com/a/679281291_120013129

RT360100-4:

Rest assured that childcare is convenient and accessible - Qingshan Lake district carried out childcare service publicity month activities.

https://ncqsh.nc.gov.cn/ncqsh/bmdt/202407/23aef7d6b89947b2a1e7681f18b30780.shtml

RT360100-5 - RT360100-6:

2024 national childcare service awareness month | Nanchang city conducts series activities. https://www.tuoyupt.com/ztydetail/2638.html

RT360100-7:

Reliable childcare ·accessible and convenient - 2024 Nanchang Xihu district childcare service awareness month launches.

http://pc.yun.jxntv.cn/c/ODZzQWt6UVJmWlJmb1l1OFNUNXc1Zz09.html

RT370100. Jinan, the capital of Shandong Province (9 reported promotional activities in total)

RT370100-1:

Huaiyin district carries out "inclusive childcare, joint action" childcare service awareness month activities.

https://baijiahao.baidu.com/s?id=1768825760830085674&wfr=spider&for=pc

RT370100-2:

Shunhua Road street in the High-tech zone carried out childcare service publicity month activities. https://dzrb.dzng.com/articleContent/30_1149159.html

RT370100-3:

http://jnfpa.jinan.gov.cn/art/2022/7/11/art_3306_294147.html

RT370100-4:

http://jnmhc.jinan.gov.cn/art/2022/5/23/art_14364_4789335.html

RT370100-5 - RT370100-7:

Fengcheng street, Laiwu district, Jinan city, carried out the centralized publicity activity of "assured childcare, convenient and accessible" childcare service publicity month.

https://jidong.sdnews.com.cn/lwqx/202407/t20240718_4412614.htm

RT370100-8:

Qianfoshan community Family Planning Association carried out childcare service publicity month activities. https://www.ql1d.com/general/24219124.html

RT370100-9:

Xingfu Street will carry out the 2024 childcare service publicity month and the "Spring Eugenics" series of publicity activities.

https://jinan.dzwww.com/qcxw/hyq/202407/t20240704_14440435.htm

RT370200. Qingdao, a sub-provincial city in Shandong Province (10 reported promotional activities in total)

RT370200-1:

Through the lens we revisit these wonderful and beautiful moments of Shibei district's childcare service awareness month. https://www.sohu.com/a/691457867_121106991

RT370200-2:

Qingdao infant and toddler care service policy promotion month launches "childcare services in progress" series feature program.

https://mp.weixin.qq.com/s?__biz=MjM5MzkxMTQ3MA==&mid=2650532847&idx=6&sn=5c9eb7983ebec230d6facb3af70eb209&chksm=be808c2e89f70538a8c6a91f248238b3ac90c46ecc965d328b21c737d46c49979d07a5b8f4f0&scene=27

RT370200-3:

Jiaozhou carries out intensive promotion activities on infant and toddler care service policies. https://www.sohu.com/a/546684921_121123738

RT370200-4:

"Childcare services in progress, health science enters communities" series activities enter Shibei district Xinglong road street.

https://mp.weixin.qq.com/s?__biz=MjM5NzIwNDQyMQ==&mid=2650781493&idx=2&sn=13565fdb8143811265478b13ad49b1a8&chksm=bed68f9f89a10689ab28a1c9c243a4a9d3f8bb36bbdbb67db6151d52a4d41c21fa12d68ac7e0&scene=27

RT370200-5:

Shibei district holds "childcare services in progress" health science community entry activity.

http://www.qingdaoshibei.gov.cn/bmxxgkmlx_9/qwjj_9/xxgkml_9/ywxx_9/202304/t20230414_7116756.shtml

RT370200-6:

"Medical" supporting health, "parenting" accompanying growth - Licang district holds "childcare services in progress" health science community entry and "medical-parenting alliance" launch ceremony.

http://wsjkw.qingdao.gov.cn/ywfl/jcfy/jcws/202306/t20230609_7221014.shtml

RT370200-7:

Shibei district Dengzhou road street carries out childcare service promotion activities entering communities. https://qd.ifeng.com/c/8GpcMiPQUVe

RT370200-8:

Qingdao Family Planning Association "5.29 member day" and infant and toddler care service policy promotion month launch ceremony is held in Chengyang district.

http://wsjkw.qingdao.gov.cn/ywdt/gzdt/202205/t20220526_6074978.shtml

RT370200-9:

Laixi city Health Commission organizes and conducts "childcare services in progress, health science enters communities" promotion campaign.

http://www.laixi.gov.cn/xxgk_16/bmxxgkml_16/lxswsjkj_16/xxgkml_16/gzdt_16/202410/t20241008_8372256.shtml

RT370200-10:

Building dreams with craftsmanship, setting sail with ambition - 2024 Qingdao childcare service awareness month launch ceremony is grandly held in Licang district.

https://mp.weixin.qq.com/s?__biz=MzAwODYxNDIxNg==&mid=2650514656&idx=1&sn=22c47c5edef1b04d8b234bb03c06852c&chksm=82984dcad09199c55504ad84308020e38eb5f757e7aae9302be78987c84ba7eecb2e8fe5a48c&scene=27

RT410100. Zhengzhou, the capital of Henan Province (20 reported promotional activities in total)

RT410100-1:

The Central Plains district Health Commission held a series of publicity activities to optimize fertility policies. https://www.zhongyuan.gov.cn/bmdt/8003676.jhtml

RT410100-2:

27 sessions in 18 cities! highlights of Henan province infant and toddler care service awareness month activities for children under 3 years old.

https://www.sohu.com/a/410945470_99964633

RT410100-3:

Zhengdong new district carried out publicity activities for infant care services.

https://baijiahao.baidu.com/s?id=1673799152726080506&wfr=spider&for=pc

RT410100-4 - RT410100-6:

Supporting childcare development, safeguarding healthy growth of infants and toddlers - Zhengdong new district deeply carries out superior birth and parenting promotion activities into ten thousand households. https://wap.zhengguannews.cn/html/zgh/139895.html

RT410100-7:

Longwang sub-district office conducted a promotional campaign on childcare services within its jurisdiction. https://www.zzhkgq.gov.cn/2023/10-20/2894662.html

RT410100-8:

https://www.163.com/dy/article/HEGBDK1Q0552ADWT.html

RT410100-9:

Zhangzhuang sub-district office carries out "promoting childcare service development, nurturing healthy growth of infants and toddlers" promotion campaign.

https://m.zzhkgq.gov.cn/2023/03-31/2550190.html

RT410100-10:

Zhengzhou: "mom's classroom" explores new childcare models to support healthy development of infants and toddlers. https://city.dahe.cn/2023/03-02/1196291.html

RT410100-11:

Zhengzhou launches Henan childcare awareness month: true love early childhood wins parents' hearts. https://www.sohu.com/a/410564537_99964633

RT410100-12:

"Nurturing infants, nurturing love" — Daxue road sub-district in Erqi District, Zhengzhou, held a thematic awareness campaign on infant and toddler care services.

https://www.sohu.com/a/409275614_100126641

RT410100-13:

https://wjw.zhengzhou.gov.cn/zwxx/3525263.jhtml

RT410100-14:

Zhengzhou High-tech zone Wutong sub-district office actively carried out a thematic campaign on "childcare services for children under 3".

http://gov.hnr.cn/hkt/article/1/1284063743076077568

RT410100-15 - RT410100-16:

Yuxing Road sub-district office launched a series of promotional activities for "standardizing childcare services central China tour".

https://wap.zhengguannews.cn/html/zgh/173652.html

RT410100-17:

Conducting public welfare activities to safeguard children's growth.

https://zzrb.zynews.cn/html/2023-06/15/content_1381691.htm

RT410100-18:

https://v.douyin.com/urchL_1hmwE

RT410100-19:

Henan province's 2024 "enhancing childcare service quality awareness month" campaign was launched at the third affiliated hospital of Zhengzhou University.

https://y.dxy.cn/hospital/50/955363.html

RT410100-20:

Reliable childcare, accessible and convenient! 2024 Huiji district childcare service awareness month launches. https://news.dahe.cn/2024/06-17/1772445.html

RT420100. Wuhan, the capital of Hubei Province (10 reported promotional activities in total)

RT420100-1:

Making parenting easier! Qingqing education childcare services are by your side.

https://www.sohu.com/a/733301432_121124620

RT420100-2:

Wuhan city holds the 25th "5.29" Family Planning Association member day commemorative event and "childcare service awareness month" promotion campaign.

https://mp.weixin.qq.com/s?__biz=MzI0NzgyNzc1OQ==&mid=2247516726&idx=2&sn=08d115886bb408d3fa1317ee10bffab6&chksm=e9a8de76dedf5760320b037134e02f871e69435ed13ad9698cd27973bc4be4dc7f3666859d36&scene=27

RT420100-3:

Hongshan district, Wuhan launches the 2024 national childcare service awareness month initiation ceremony.

https://baijiahao.baidu.com/s?id=1803546683566477434&wfr=spider&for=pc

RT420100-4:

Healthy families, bright future - inclusive childcare policy reaches Hanyang.

https://m.163.com/dy/article/JIBJTETC05346936.html

RT420100-5:

Jiangxia district, Wuhan carries out "childcare service awareness month" series activities. https://news.hubeidaily.net/hbrbsharenew/news_detail/5/2834026/2559086/0

RT420100-6:

Wuhan: "reliable childcare, accessible and convenient" - Wuhan city childcare service awareness month campaign begins.

https://wjw.hubei.gov.cn/bmdt/szsm/202407/t20240703_5258289.shtml

RT420100-7:

"Reliable childcare, accessible and convenient" - Jiufeng street, East Lake High-tech Zone conducts childcare service awareness month activities.

http://www.hubeitoday.com.cn/post/34/189093

RT420100-8:

Central Garden community organizes childcare service awareness month campaign. http://www.hubeitoday.com.cn/post/34/188055

RT420100-9:

Caijiazhai street carries out 2024 childcare service awareness month activities.

http://hbwh.wenming.cn/oldweb/hp/202407/t20240704_8588326.html

RT420100-10:

Qingshan district conducts "reliable childcare, accessible and convenient" 2024 childcare service awareness month campaign.

https://www.qingshan.gov.cn/qzfbm/zfgzbm/wsj/bmdt/202407/t20240708_2425551.shtml

RT430100. Changsha, the capital of Hunan Province (7 reported promotional activities in total)

RT430100-1:

Scientific parenting promotes growth - Changsha's "scientific parenting into ten thousand homes" enters Yuhua district.

https://baijiahao.baidu.com/s?id=1777343231917068534&wfr=spider&for=pc

RT430100-2:

"Scientific parenting into ten thousand homes" public welfare activity enters Mawangdui kindergarten. https://baijiahao.baidu.com/s?id=1777838871724305300&wfr=spider&for=pc

RT430100-3:

Joyful gathering, "parenting" gifts: Hunan maternal and child health hospital hosts loving toddler care event.

https://health.rednet.cn/m/content/646845/68/13473953.html

RT430100-4:

Scientific parenting into ten thousand homes, we are in action.

https://www.csysgz.com/info/1208/12523.htm

RT430100-5:

"Toddler care" for all: Furong district hosts "scientific parenting for every family" charity initiative.

http://www.furong.gov.cn/affairs/fdzdgknr/zdms/gysy/202309/t20230911_11215885.html

RT430100-6:

Jinzhi Weiye appears at Hunan province 2023 childcare service awareness month launch ceremony. https://www.163.com/dy/article/I74GDDC50552NTB0.html

RT430100-7:

The 2024 national childcare service awareness month kicks off in Changsha.

https://www.hunan.gov.cn/hnszf/hnyw/szdt/202406/t20240616_33328338.html

RT440100. Guangzhou, the capital of Guangdong Province (7 reported promotional activities in total)

RT440100-1:

Baiyun district, Guangzhou launches childcare service awareness month campaign. https://baijiahao.baidu.com/s?id=1769467563315383737&wfr=spider&for=pc

RT440100-2:

"Inclusive childcare ·collective action" - Huadu district conducts voluntary childcare service promotion activities.

https://mp.weixin.qq.com/s?__biz=MzAwNDAzMjA1Mg==&mid=2649926666&idx=2&sn=712fc5cf92379d63ef163beb0623c9a9&chksm=83341f51b4439647009f70120b55a250d234571b72a5dcd32f4f83ede6611dd9ad380c131463&scene=27

RT440100-3:

"Inclusive childcare, joint action" Zhujiang street carries out childcare service awareness month activities.

https://www.gzns.gov.cn/zfxxgkml/gzsnsqzjjdbsc/zwdt/content/post_9062740.html

RT440100-4:

Panyu district holds childcare service awareness month event.

https://www.panyu.gov.cn/gkmlpt/content/9/9057/mpost_9057082.html#7524

RT440100-5:

"Worry-free childcare" – Guangzhou officially releases mini-documentary on childcare services. https://tuoyupt.com/ztydetail/2633.html

RT440100-6:

Conghua district, Guangzhou successfully concludes community training and awareness campaign on childcare services. https://www.sohu.com/a/843541103_121713508

RT440100-7:

Launch of the 2024 Guangzhou Conghua district "Nanyue home services" Yangcheng action childcare project scientific parenting training initiative.

https://www.toutiao.com/article/7450055422276305419/

RT440300. Shenzhen, a sub-provincial city in Guangdong Province (2 reported promotional activities in total)

RT440300-1:

Reliable childcare, accessible and convenient - our kindergarten actively responds to the invitation and participates in Shenzhen childcare service awareness month activities.

https://mp.weixin.qq.com/s?__biz=MzA4MDY1NzcxNw==&mid=2649753738&idx=1&sn=462f3457db3e61ceab02d4994570861d&chksm=87a444d0b0d3cdc61d89a2971f74e296752ffe0899ab739459349d14ad8f2ff0905e42a21343&scene=27

RT440300-2:

Lianhua street holds "quality nurturing for the young, quality education for learners" scientific parenting promotion campaign.

https://baijiahao.baidu.com/s?id=1804658055896810919&wfr=spider&for=pc

RT450100. Nanning, the capital of Guangxi Zhuang Autonomous Region (14 reported promotional activities in total)

RT450100-1:

https://wjw.nanning.gov.cn/gzdt/xqdt/t5749522.html

RT450100-2:

Qingxiu district deploys 100 promotional displays to enhance awareness rate of childcare service policies. http://www.qingxiu.gov.cn/zhuanti/ywzt/rkjt/tyzh/t5634655.html

RT450100-3:

Nanning Qingxiu district carries out infant and toddler care service promotion month thematic activities for 0-3 year olds. http://nnwb.nnnews.net/p/44672.html

RT450100-4:

https://k.sina.com.cn/article_3177450665_bd640ca9020015pui.html

RT450100-5:

http://www.nnnews.net/xianqu/p/3079659.html

RT450100-6:

https://wjw.nanning.gov.cn/gzdt/xqdt/t5812987.html

RT450100-7:

https://wjw.nanning.gov.cn/gzdt/xqdt/t4782842.html

RT450100-8:

https://wjw.nanning.gov.cn/gzdt/xqdt/t4745214.html

RT450100-9:

https://wjw.nanning.gov.cn/gzdt/xqdt/t4776961.html

RT450100-10:

https://wjw.nanning.gov.cn/gzdt/wsdt/t5596326.html

RT450100-11:

https://wjw.nanning.gov.cn/gzdt/xqdt/t4764777.html

RT450100-12 - RT450100-13:

"Reliable childcare, accessible and convenient" - Nanning maternal and child health hospital conducts childcare service awareness month campaign.

https://wjw.nanning.gov.cn/gzdt/t5972000.html

RT450100-14:

Binyang county carries out 2024 childcare service promotion activities.

https://wjw.nanning.gov.cn/gzdt/t5933421.html

RT460100. Haikou, the capital of Hainan Province (3 reported promotional activities in total)

RT460100-1:

National childcare awareness month parent-child activity was held at Haikou Longhua district cultural center. http://www.hkwb.net/news/content/2023-06/04/content_4215059.htm

RT460100-2:

2024 Hainan province childcare service awareness month launch ceremony was successfully held.

https://mp.weixin.qq.com/s?__biz=MzA3NzIwMjkxNQ==&mid=2649578227&idx=1&sn=58c5764a5b7d11d8bd1d923beb6bb511&chksm=86dad6266792c6e596e9cd55bcaa4229c7c725cf8b81b9b72ef01fbe496d6917ccfdd4f89d55&scene=27

RT460100-3:

Haikou Xiuying district held the 2024 childcare service awareness month and World Population Day promotion event.

https://www.hinews.cn/news/system/2024/07/12/033177576.shtml

RT500000. Chongqing, city (9 reported promotional activities in total)

RT500000-1:

Protecting maternal and child health - Bishan district carries out the 34th World Population Day series promotion activities. https://www.163.com/dy/article/I9RTVL9905346936.html

RT500000-2:

Caiyuanba street conducts 2023 childcare service month promotion campaign.

https://www.cqyz.gov.cn/jz_229/cybjdbsc/zwxx_97154/dt/202306/t20230630_12108380.html

RT500000-3:

Fuling carries out "inclusive childcare, joint action" series promotion activities.

http://www.fl.gov.cn/zwxx_206/ywdt/202306/t20230612_12054280.html

RT500000-4:

Jiangbei holds "childcare open day" event.

https://baobao.sohu.com/a/737306955_120578424

RT500000-5:

Dianjiang county launches 2023 national childcare service awareness month campaign.

http://www.cq.xinhuanet.com/2023-06/02/c_1129665727.htm

RT500000-6:

Liangjiang new area establishes infant and toddler care guidance center to promote high-quality childcare development.

https://www.liangjiang.gov.cn/mixmedia/a/202407/11/WS668f996de4b0541d1b296002.html

RT500000-7:

Nan'an district 2024 national childcare service awareness month and World Population Day promotion campaign kicks off.

http://www.cqna.com.cn/na_content/2024-07/05/content_10700337.html

RT500000-8:

2024 Liangping district childcare service awareness month activities are held.

https://www.cqlprm.cn/news-center/detail/99732363?sourceType=1

RT500000-9:

Jiangjin district holds 2024 national childcare service awareness month and optimized birth policy service project launch ceremony.

https://baijiahao.baidu.com/s?id=1804202165191571437&wfr=spider&for=pc

RT510100. Chengdu, the capital of Sichuan Province (4 reported promotional activities in total)

RT510100-1:

Inclusive childcare, Longquan in action - Longquan street carried out the 2023 childcare service awareness month campaign. https://news.sohu.com/a/681610706_121106832

RT510100-2:

Nurturing healthy growth of infants and toddlers - Wenjiang district conducted childcare awareness thematic activities. https://new.qq.com/rain/a/20230914A0211300

RT510100-3:

https://cdwjw.chengdu.gov.cn/cdwjw/gzdt/2020-10/19/content_2525797d56b94d60b4fd830126c08d36.shtml

RT510100-4:

National childcare service awareness month and Chengdu childcare service promotion were successfully held.

https://baijiahao.baidu.com/s?id=1804716405216328670&wfr=spider&for=pc

RT520100. Guiyang, the capital of Guizhou Province (5 reported promotional activities in total)

RT520100-1:

Yuntan street: "inclusive childcare" enters the community, making parenting no longer difficult. https://m.thepaper.cn/baijiahao_23507523

RT520100-2:

Nanming district Health Commission conducts "inclusive childcare, joint action" childcare service awareness month activities. https://weibo.com/1923969725/N3TxwCtyt

RT520100-3:

Reliable childcare ·accessible and convenient! Guiyang Guanshanhu district launches 2024 childcare service awareness month campaign.

http://www.gzswssy.com/item/show/16-7571-1.shtml

RT520100-4:

Active actions, jointly promoting childcare development | our teachers and students participated in Qianxinan prefecture and Xingyi city's "national childcare service awareness month" event. https://www.zhijiao.cn/news/detail/183699

RT520100-5:

Guizhou province's "childcare service awareness month" and "5.29 Family Planning Association member day" promotion activities were held in Taijiang.

http://news.gog.cn/system/2024/05/17/018568068.shtml

RT530100. Kunming, the capital of Yunnan Province (2 reported promotional activities in total)

RT530100-1:

Kunming city implements multiple measures to promote childcare services for children under 3 years old.

https://baijiahao.baidu.com/s?id=1804975948605169525&wfr=spider&for=pc

RT530100-2:

Inclusive childcare joint action - Yunnan province childcare service awareness month public service video. https://news.yunnan.cn/system/2023/06/08/032621595.shtml

RT610100. Xi'an, the capital of Shaanxi Province (11 reported promotional activities in total)

RT610100-1:

Xixian new area carries out childcare service scientific parenting promotion activities. http://xawjw.xa.gov.cn/gzdt/qxdt/6502c3adf8fd1c1a7038fe4b.html

RT610100-2:

Xi'an city intensively conducts infant and toddler care service knowledge awareness month activities. http://xawjw.xa.gov.cn/gzdt/wjyw/1707314195833331714.html

RT610100-3:

Xi'an city launches infant and toddler care service knowledge awareness month campaign. http://xawjw.xa.gov.cn/gzdt/wjyw/6476b870f8fd1c1a7030246f.html

RT610100-4:

http://xawjw.xa.gov.cn/gzdt/qxdt/61974a0bf8fd1c0bdc6b3042.html

RT610100-5:

http://xawjw.xa.gov.cn/gzdt/qxdt/62834d02f8fd1c0bdc984473.html

RT610100-6:

"Live up to the trust, nurture with love" - Xixian new area initiates childcare service

awareness month activities.

https://baijiahao.baidu.com/s?id=1767032277837750173&wfr=spider&for=pc

RT610100-7:

Xi'an Beilin district Wenyi road street Family Planning Association holds "promoting childcare service development, nurturing healthy growth of infants and toddlers" 5.29 member activity month promotion event.

https://mp.weixin.qq.com/s?__biz=MzI3NTU1OTQ4OA==&mid=2247505449&idx=2&sn=e89c6c3cd7fc2e644fd8380aab12c82e&chksm=eb007306dc77fa108d13797b8d2a021fbed20914c740bad9ff626d1f6c6e7db7a36a00de086e&scene=27

RT610100-8:

Xi'an Huyi district organizes childcare service awareness thematic activities.

http://gov.cnwest.com/zwyw/a/2023/08/14/21786653.html

RT610100-9:

Scientific parenting, we are in action.

http://www.baqiao.gov.cn/xwzx/gzdt/65041f6df8fd1c1a703914d7.html

RT610100-10:

Reliable childcare, accessible and convenient - Baishulin street conducts childcare promotion campaign.

http://www.beilin.gov.cn/xwzx/bmdt/1809154521729425410.html

RT610100-11:

http://www.xixianxinqu.gov.cn/xwzx/jzdt/1812666489878700033.html

RT620100. Lanzhou, the capital of Gansu Province (5 reported promotional activities in total)

RT620100-1:

Qilihe district organizes and conducts promotion activities for private childcare institutions. https://baijiahao.baidu.com/s?id=1774194602384921457&wfr=spider&for=pc

RT620100-2:

Nurturing healthy growth of infants and toddlers - our province holds childcare service promotion campaign.

https://baijiahao.baidu.com/s?id=1767020085942036155&wfr=spider&for=pc

RT620100-3:

Inclusive childcare, joint action - Anning district childcare service awareness month kicks off. https://weibo.com/5813728966/N40Jx9C41

RT620100-4:

Inclusive childcare joint action cultural performance and promotion activity commences at Lanzhou Peili square.

https://baijiahao.baidu.com/s?id=1767729161656905925&wfr=spider&for=pc

RT620100-5:

"Reliable childcare, accessible and convenient" - our province childcare service awareness month campaign launches.

https://www.lzbs.com.cn/zbxw/2024-06/16/content_506443734.htm

RT630100. Xining, the capital of Qinghai Province (3 reported promotional activities in total)

RT630100-1:

Reliable childcare, accessible and convenient - childcare service awareness month series activities.

https://mp.weixin.qq.com/s?__biz=MzI4NjA1MTAzOA==&mid=2657497330&idx=6&sn=ef4157049d770565ee650e7c641d714c&chksm=f0704f43c707c65533719c3919c2aca9b44ed45b26e2b2274e33b2bd44d941f2364073f862c4&scene=27

RT630100-2:

Vigorously developing childcare services to promote balanced population development - provincial childcare service awareness month and "7.11" World Population Day large-scale promotion campaign.

https://mp.weixin.qq.com/s?__biz=MzI0MTU4Nzg0NQ==&mid=2247530641&idx=1&sn=be6e1d656b1fedcfe2a9f82d81e70a7f&chksm=e90b7b7bde7cf26dc9211d4179c30afaee06a5d965f4bd6d65af6e4c09a99aeb53e1ed2e39ba&scene=27

RT630100-3:

Difficult parenting? let childcare institutions help you.

https://wjw.xining.gov.cn/sy/ztzl/jkxn/202407/t20240730_206162.html

RT640100. Yinchuan, the capital of Ningxia Hui Autonomous Region (3 reported promotional activities in total)

RT640100-1 - RT640100-3:

Summary of the childcare service awareness month by Jinfeng district Health Commission.

http://www.ycjinfeng.gov.cn/xxgk/xxgkbm/jfqwjj/xxgkml_19063/ylws_19068/202308/t20230815_4219366.html

RT650100. Urumqi, the capital of Xinjiang Uygur Autonomous Region (1 reported promotional activity in total)

RT650100-1:

Xinjiang launches childcare service awareness month campaign.

https://wjw.xinjiang.gov.cn/hfpc/xwxc1/202406/75ed25eccd9443f8bb6e1455e1876449.shtml
